# Supplementary material for: MECP2 mutations affect ciliogenesis: a novel perspective for Rett syndrome and related disorders
Source: EMBO Mol Med. 2020 May 8;12(6):e10270. doi: 10.15252/emmm.201910270 (PMC7278541; doi:10.15252/emmm.201910270)

Table of content of the Appendix

- **Appendix table S1**.
- **Appendix figure S1**.

Appendix table S1 P value and the statistical test used for each comparison are indicated for each figure.

**Figure 1**	**P value**	**Comparison**	**Statistical test**
1B	0.0043	WT vs *Mecp2* null	Mann-Whitney test
1C	0.0001	WT vs *Mecp2* null	Student's t test
1F	0.0001	CTRL vs siMeCP*2*	Student's t test
1G	0.001	CTRL vs siMeCP*2*	Mann-Whitney test
1H	0.0007	CTRL vs siMeCP*2*	Student's t test
1J	0.0001	WT vs *Mecp2* null at DIV3	Two-way ANOVA, Bonferroni post hoc test
	0.0001	WT vs *Mecp2* null at DIV7	Two-way ANOVA, Bonferroni post hoc test
	0.0001	WT vs *Mecp2* null at DIV14	Two-way ANOVA, Bonferroni post hoc test
1K	0.0057	WT vs *Mecp2* null at DIV3	Two-way ANOVA, Bonferroni post hoc test
	0.0001	WT vs *Mecp2* null at DIV7	Two-way ANOVA, Bonferroni post hoc test
	0.0028	WT vs *Mecp2* null at DIV14	Two-way ANOVA, Bonferroni post hoc test
1M	0.0151	WT vs *Mecp2* null	Student's t test
1N	0.0001	WT vs *Mecp2* null	Student's t test
**Figure 2**	**P value**	**Comparison**	**Statistical test**
2C	0.0001	WT+GFP vs *Mecp2* null+GFP	Two-way ANOVA, Bonferroni post hoc test
	0.0001	*Mecp2* null+GFP vs *Mecp2* null+MeCP2-iresGFP	Two-way ANOVA, Bonferroni post hoc test
2D	0.0001	WT+GFP vs *Mecp2* null+GFP	Two-way ANOVA, Bonferroni post hoc test
	0.0072	*Mecp2* null+GFP vs *Mecp2* null+MeCP2-iresGFP	Two-way ANOVA, Bonferroni post hoc test
**Figure 3**	**P value**	**Comparison**	**Statistical test**
3B	0.0001	CTRL vs siMeCP*2* at 200nM	Two-way ANOVA, Bonferroni post hoc test
	0.0001	CTRL vs siMeCP*2* at 500nM	Two-way ANOVA, Bonferroni post hoc test
3C	0.01	WT vs *Mecp2* null at 500mM	Two-way ANOVA, Bonferroni post hoc test

3D	0.0009	WT UT vs WT SAG	Two-way ANOVA, Bonferroni post hoc test
	0.0012	WT SAG vs *Mecp2* null SAG	Two-way ANOVA, Bonferroni post hoc test
3E	0.0003	WT UT vs WT SAG 400nM	Two-way ANOVA, Bonferroni post hoc test
	0.0322	WT SAG 400nM vs *Mecp2* null SAG 400 nM	Two-way ANOVA, Bonferroni post hoc test
3F	0.0029	WT SAG vs *Mecp2* null SAG	Two-way ANOVA, Bonferroni post hoc test
**Figure 4**	**P value**	**Comparison**	**Statistical test**
4B	0.0159	WT vs *Mecp2* null	Mann-Whitney test
4C	0.001	WT vs *Mecp2* null Layer1	Two-way ANOVA, Bonferroni post hoc test
	0.0018	WT vs *Mecp2* null Layer2/3	Two-way ANOVA, Bonferroni post hoc test
	0.0149	WT vs *Mecp2* null Layer4	Two-way ANOVA, Bonferroni post hoc test
4E	0.0001	WT vs *Mecp2* null IGL	Mann-Whitney test
	0.0001	WT vs *Mecp2* null EGL	Mann-Whitney test
4F	0.0021	WT vs *Mecp2* Het IGL	Mann-Whitney test
	0.0003	WT vs *Mecp2* Het EGL	Mann-Whitney test
4G	0.0425	WT vs *Mecp2* null	Student’s t test
4H	0.0426	WT vs *Mecp2* null (Gli1)	Student’s t test
	0.0435	WT vs *Mecp2* null (CycD1)	Student’s t test
**Figure 5**	**P value**	**Comparison**	**Statistical test**
5A	0.0015	WT UT vs *Mecp2* null UT	Two-way ANOVA, Bonferroni post hoc test
	0.044	WT VEH vs *Mecp2* null VEH	Two-way ANOVA, Bonferroni post hoc test
	0.0047	*Mecp2* null UT vs *Mecp2* null+tubacin	Two-way ANOVA, Bonferroni post hoc test
	0.0497	*Mecp2* null VEH vs *Mecp2* null+tubacin	Two-way ANOVA, Bonferroni post hoc test
5B	0.0001	WT UT vs *Mecp2* null UT	Two-way ANOVA, Bonferroni post hoc test
	0.0011	*Mecp2* null UT vs *Mecp2* null+tubacin	Two-way ANOVA, Bonferroni post hoc test
	0.0474	*Mecp2* null VEH vs *Mecp2* null+tubacin	Two-way ANOVA, Bonferroni post hoc test
5C	0.0001	CTRL+SAG vs siMecp2+SAG	Three-way ANOVA, Tukey’s post hoc test
	0.0001	siMecp2 SAG vs siMecp2 SAG+tubacin	Three-way ANOVA, Tukey’s post hoc test

Figure 6	P value	Comparison	Statistical test
6B	0.0025	WT UT vs *Mecp2* null UT	Mann-Whitney test
	0.0184	*Mecp2* null UT vs *Mecp2* null+tubacin	One-way ANOVA, Dunn's post hoc test
	0.0020	*Mecp2* null UT vs *Mecp2* null+TC-S7010	One-way ANOVA, Dunn's post hoc test
6D	0.0001	WT UT vs *Mecp2* null UT	Mann-Whitney test
	0.0189	*Mecp2* null UT vs *Mecp2* null+tubacin	One-way ANOVA, Dunn's post hoc test
	0.0001	*Mecp2* null UT vs *Mecp2* null+TC-S7010	One-way ANOVA, Dunn's post hoc test
6E	0.0046	WT UT vs *Mecp2* null UT	Mann-Whitney test
	0.0001	*Mecp2* null UT vs *Mecp2* null+TC-S7010	One-way ANOVA, Dunn's post hoc test
6F	0.0008	WT UT vs *Mecp2* null UT	Mann-Whitney test
	0.0394	*Mecp2* null UT vs *Mecp2* null+TC-S7010	One-way ANOVA, Dunn's post hoc test
**Figure 7**	**P value**	**Comparison**	**Statistical test**
7C	0.0001	CTRL vs 705delG	One-way ANOVA, Dunnett's post hoc test
	0.0001	CTRL vs Q244X	One-way ANOVA, Dunnett's post hoc test
	0.0001	CTRL vs R255X	One-way ANOVA, Dunnett's post hoc test
7D	0.0089	CTRL vs 705delG	One-way ANOVA, Dunnett's post hoc test
	0.0001	CTRL vs Q244X	One-way ANOVA, Dunnett's post hoc test
	0.0218	CTRL vs R255X	One-way ANOVA, Dunnett's post hoc test
7E	0.0001	CTRL UT vs 705delG UT	Two-way ANOVA, Bonferroni post hoc test
	0.0001	CTRL UT vs Q244X UT	Two-way ANOVA, Bonferroni post hoc test
	0.0001	CTRL UT vs R255X UT	Two-way ANOVA, Bonferroni post hoc test
	0.0158	Q244X UT vs Q244X+tubacin	Two-way ANOVA, Bonferroni post hoc test
	0.0381	R255X UT vs R255X+tubacin	Two-way ANOVA, Bonferroni post hoc test
**Figure EV1**	**P value**	**Comparison**	**Statistical test**
	0.0499	WT vs *Mecp2*$^{Y120D/y}$	Mann-Whitney test
**Figure EV2**	**P value**	**Comparison**	**Statistical test**
	0.0001	WT UT vs *Mecp2* null UT	Mann-Whitney test

	0.0003	*Mecp2* null UT vs *Mecp2* null+tubacin	One-way ANOVA, Dunn's post hoc test
	0.0001	*Mecp2* null UT vs *Mecp2* null+TC-S7010	One-way ANOVA, Dunn's post hoc test
**Figure EV3**	**P value**	**Comparison**	**Statistical test**
	0.0249	WT UT vs WT+tubacin	One-way ANOVA, Dunn's post hoc test

Appendix figure S1. (**A**) As already reported (Bedogni et al., 2016), *Mecp2* null neurons (DIV7) exhibit a reduced soma size with respect to the WT cells (n=23 WT and n=23 *Mecp2* null cells; ***p<0.001 by Student's t test). (**B**) Correlation analysis between soma area and cilium length in WT and *Mecp2* null neurons at DIV7. Pearson's correlation coefficient is proximal to 0 for both WT and null cells, indicating the absence of a correlation between the two variables. (**C)** Healthy and RTT fibroblasts do not manifest difference in their soma size (n=39 CTRL and n=78 RTT fibroblasts). (**D**) Correlation analysis between soma area and cilium length in CTRL and RTT fibroblasts. Pearson's correlation coefficient is proximal to 0 for all cells, indicating the absence of a correlation between the two variables.

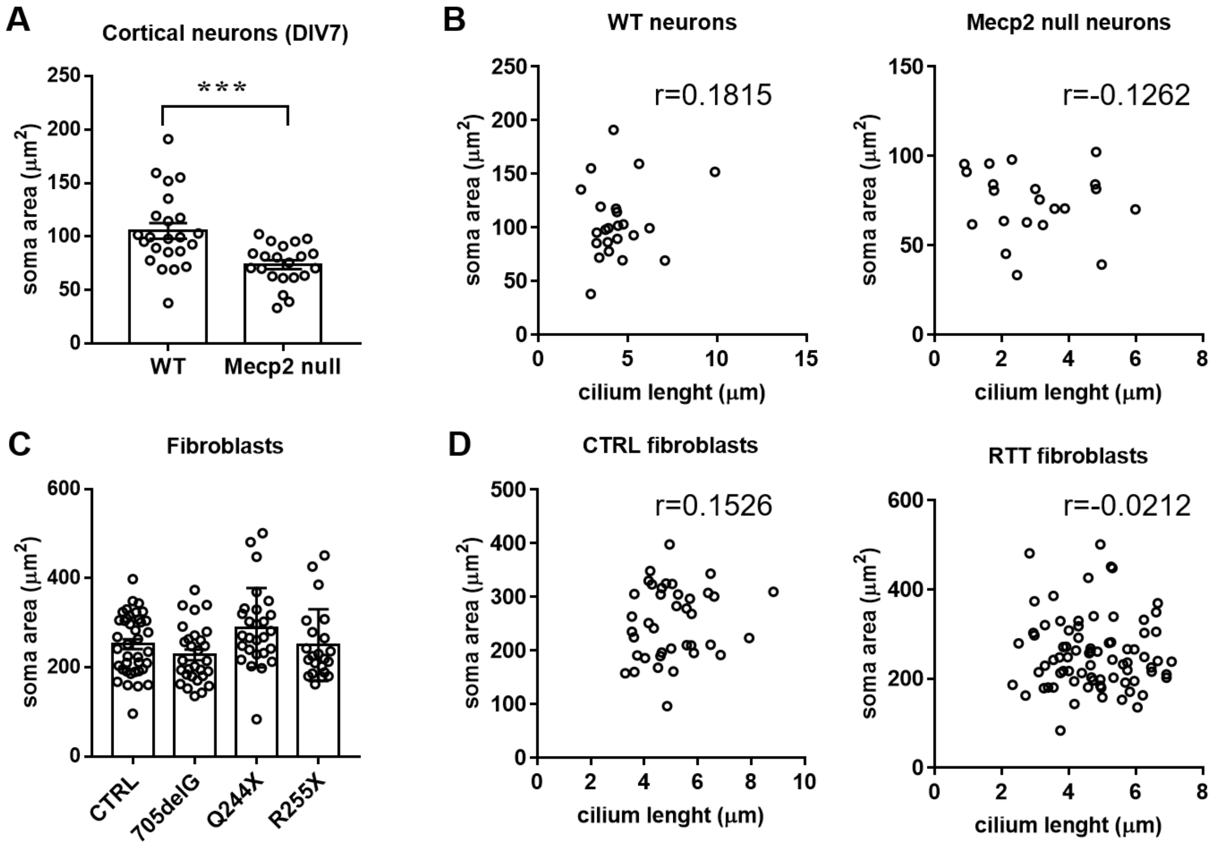

Supplement: Supplementary file 1 — Appendix [file EMMM-12-e10270-s001.pdf]
