# Supplementary material for: MECP2 mutations affect ciliogenesis: a novel perspective for Rett syndrome and related disorders
Source: EMBO Mol Med. 2020 May 8;12(6):e10270. doi: 10.15252/emmm.201910270 (PMC7278541; doi:10.15252/emmm.201910270)
Supplement: Supplementary file 3 — Expanded View Figures PDF [file EMMM-12-e10270-s002.pdf]

Expanded View Figures

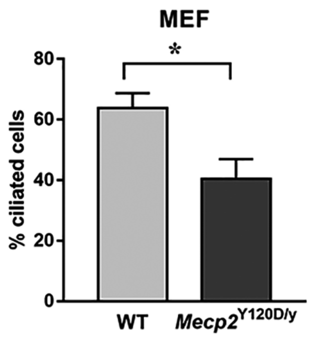

Figure EV1. Ciliogenesis is impaired in *Mecp2*$^{Y120D/y}$ murine embryonic fibroblasts (MEFs).
Immunofluorescence for acetylated γ-tubulin was performed on WT and *Mecp2*$^{Y120D/y}$ MEFs, and images were acquired under an epi-fluorescence microscope by Nikon. The graph reports the mean ± SE of the percentage of ciliated cells in WT and mutated cells, derived from three independent experiments (*$P < 0.05$, Mann–Whitney test).

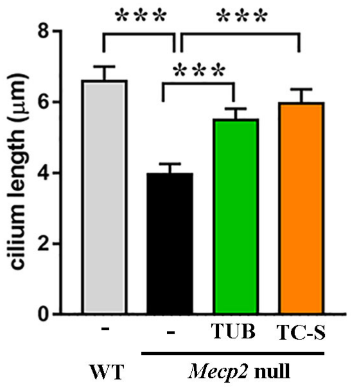

Figure EV2. Tubacin and Aurora A inhibitor elongate primary cilium in *Mecp2* null neurons.
WT and *Mecp2* null neurons (DIV5) were treated with tubacin (1 µM) or TC-S 7010 (7 nM) for 48 h or left untreated. At DIV7, neurons were fixed with 4% paraformaldehyde in PBS, and immunofluorescence for Arl13b was performed to detect primary cilium. Images from six different biological samples for each genotype were acquired, and primary cilium length was analyzed by ImageJ software ($n = 46$ WT; $n = 52$ KO; $n = 35$ KO+TUB; and $n = 51$ KO+TC-S). The graph reports the mean ± SE of the primary cilium length in untreated WT and *Mecp2* null neurons and in null cells after tubacin (TUB) or Aurora A inhibitor (TC-S) treatment. Data from WT and null neurons were compared by Mann–Whitney test (***$P < 0.001$); drug effects on null cells were analyzed by one-way ANOVA followed by Dunn's *post hoc* test (***$P < 0.001$).

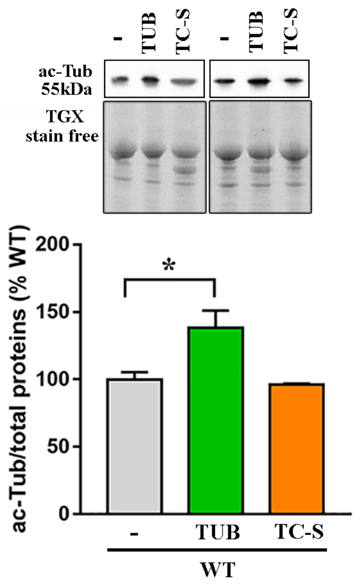

Figure EV3. Tubulin acetylation is increased by tubacin, but not by the Aurora A inhibitor.
WT neurons (DIV12) were treated with tubacin (1 µM) or TC-S 7010 (7 nM) for 48 h or left untreated. Acetylated α-tubulin (ac-Tub) was measured by Western blot in untreated and tubacin or TC-S-treated cells. Representative bands of acetylated α-tubulin are depicted above the histogram, which shows the mean ± SE of the percentage of the expression levels compared to untreated samples ($n = 3$, *$P < 0.05$, one-way ANOVA followed by Dunn's *post hoc* test). Data are normalized to total protein content, visualized by a TGX stain-free technology.

© 2020 The Authors
