# Supplementary material for: MECP2 mutations affect ciliogenesis: a novel perspective for Rett syndrome and related disorders
Source: EMBO Mol Med. 2020 May 8;12(6):e10270. doi: 10.15252/emmm.201910270 (PMC7278541; doi:10.15252/emmm.201910270)

FIGURE 1A

MEF WT

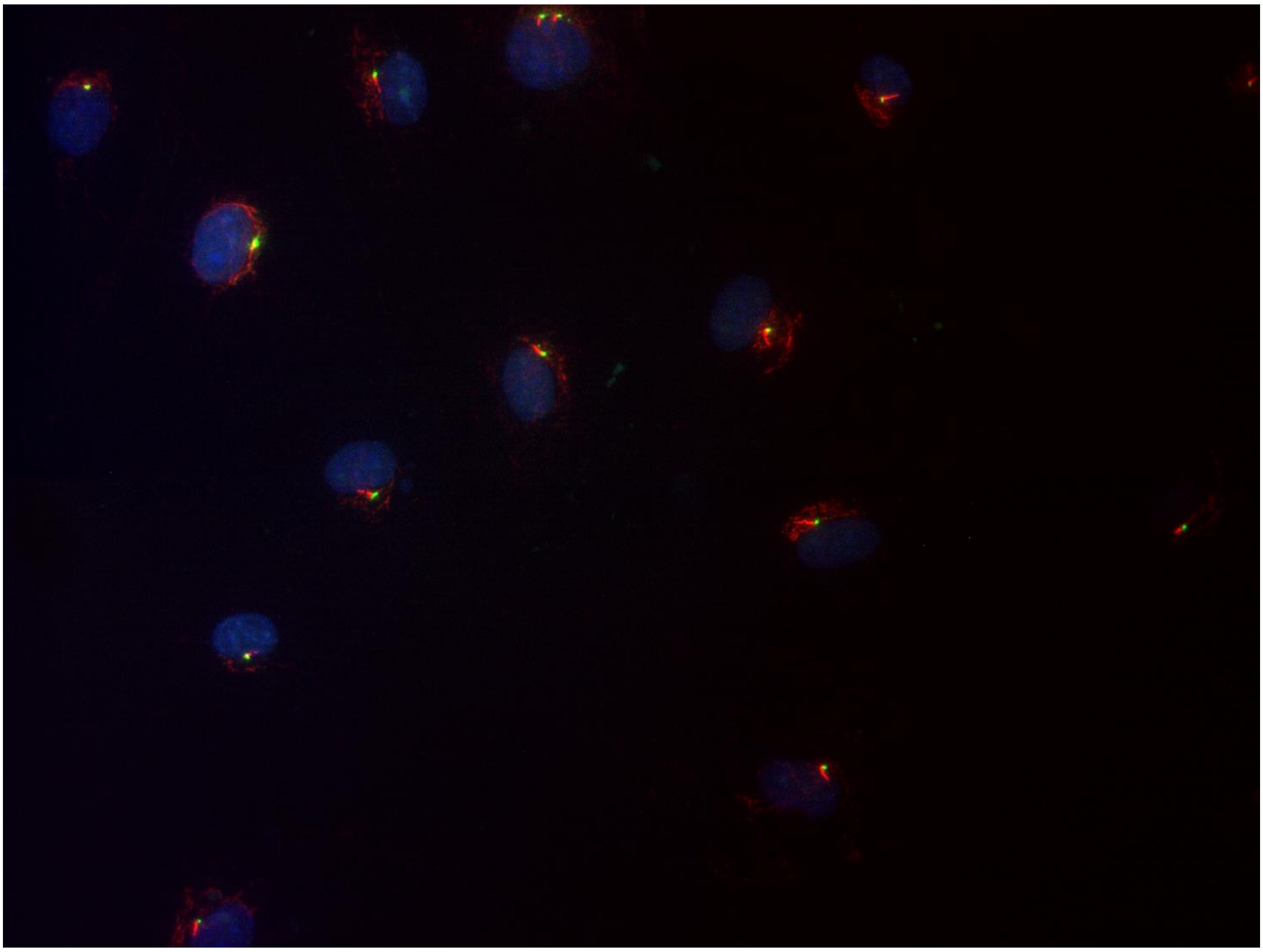

Mecp2 KO

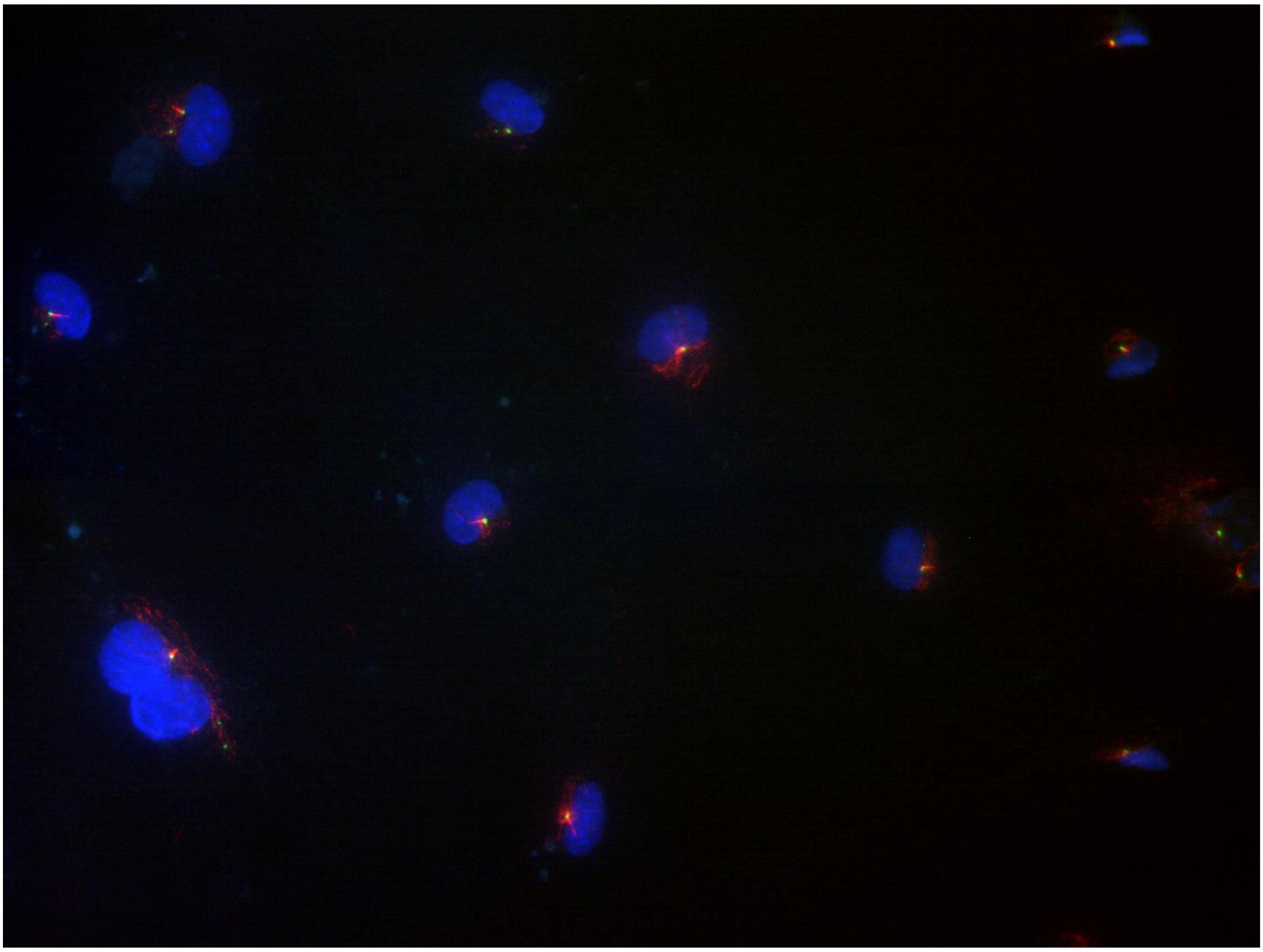

FIGURE 1D

RPE ctrl

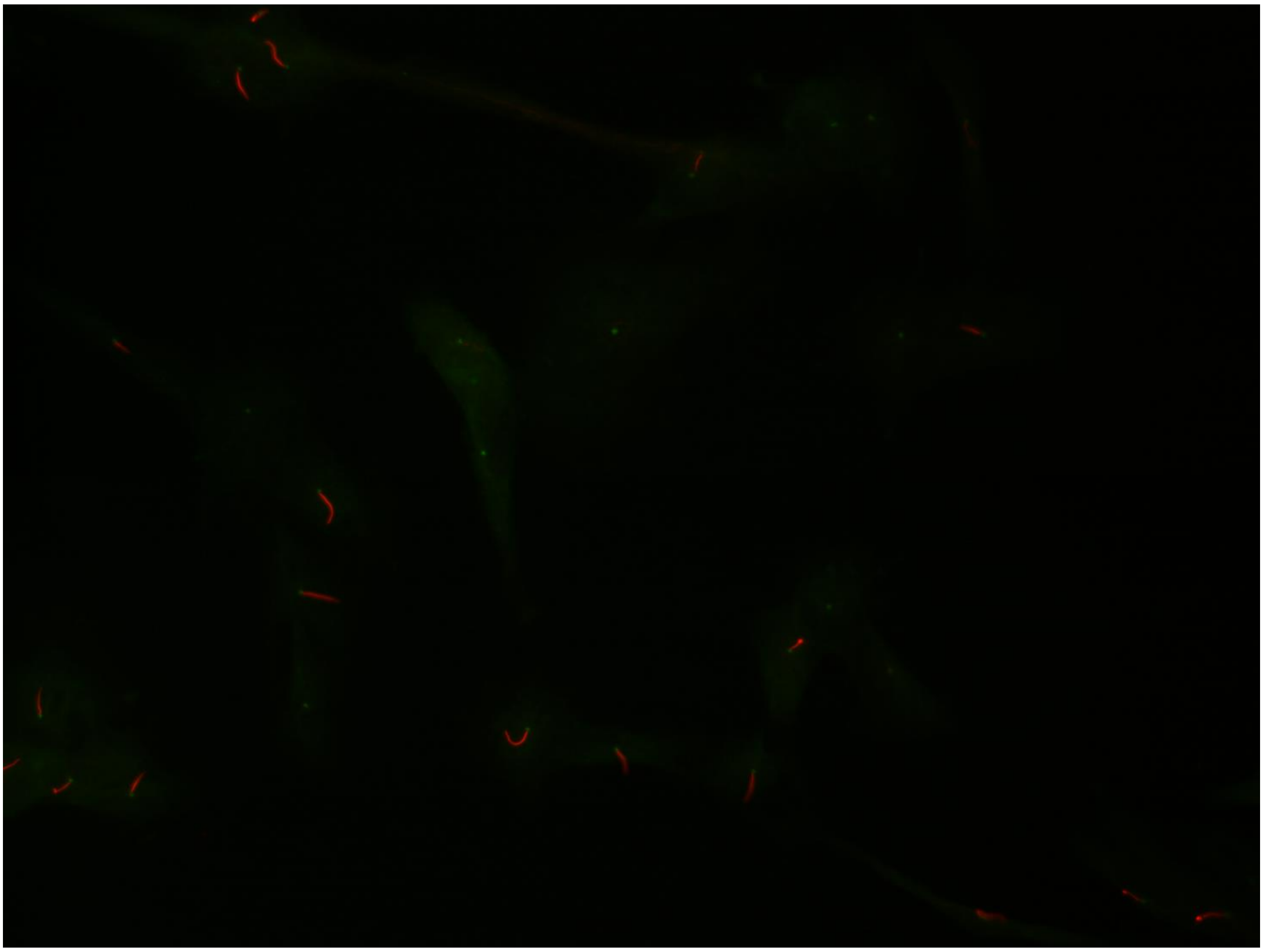

Silenced RPE

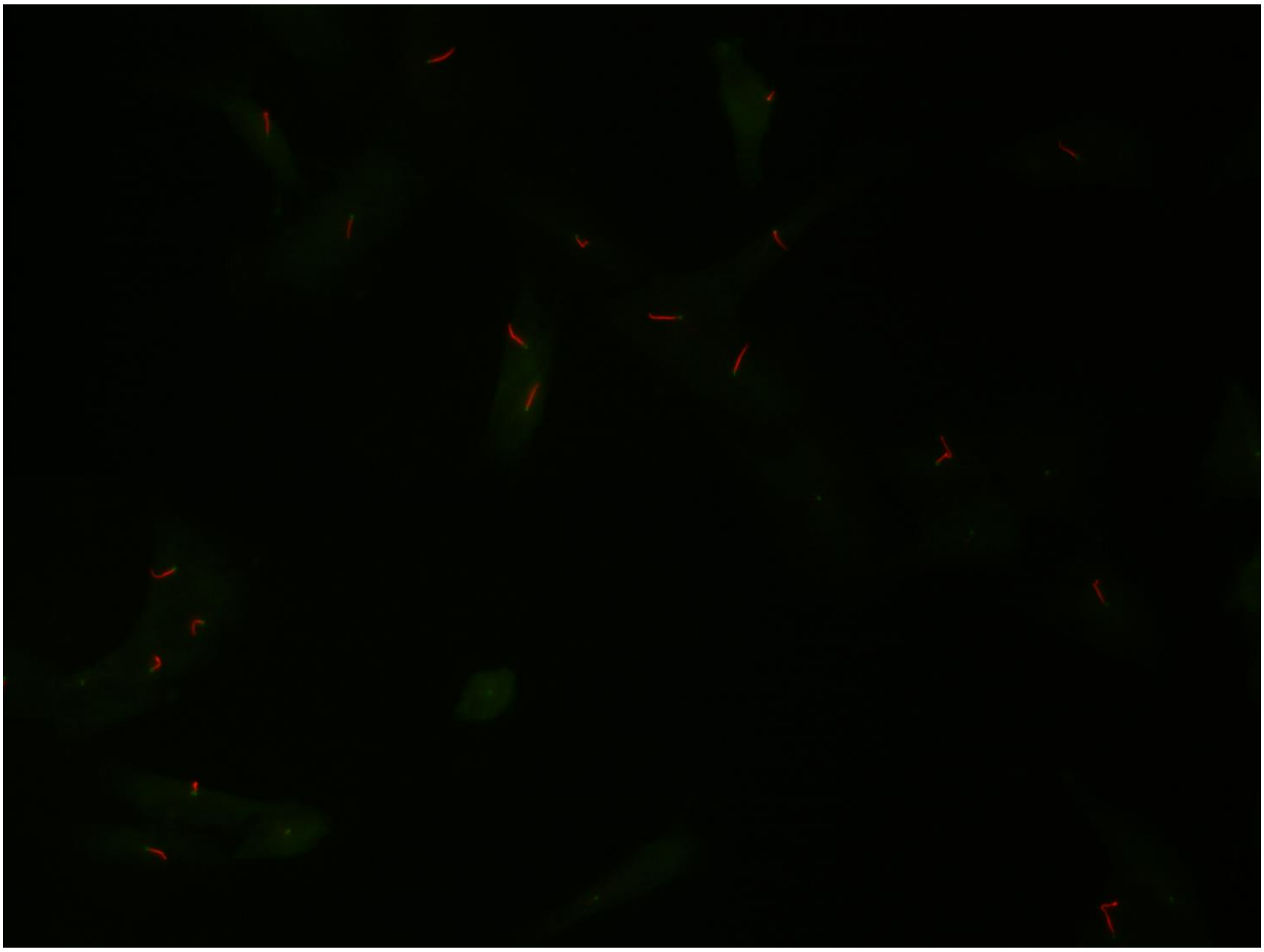

Figure 1E

Mecp2

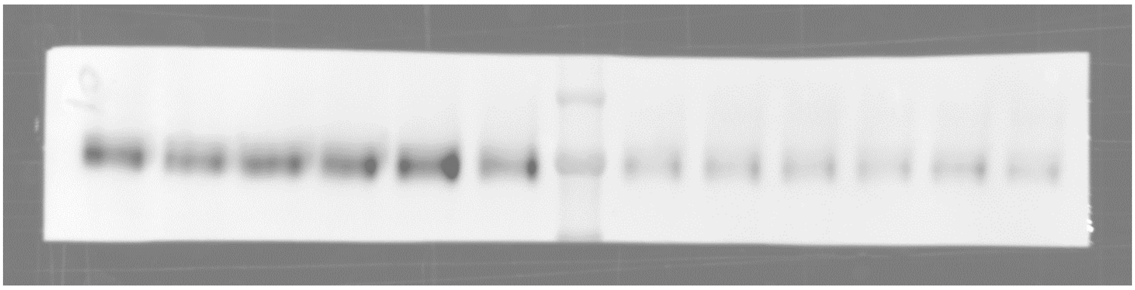

GAPDH

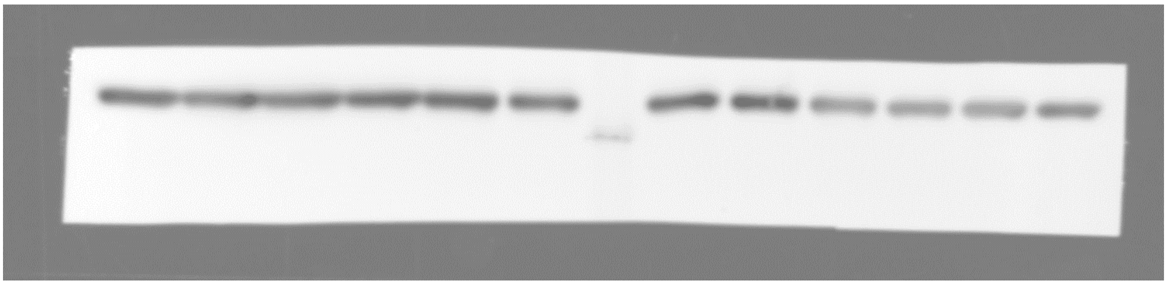

Figure 1I

WT at DIV7

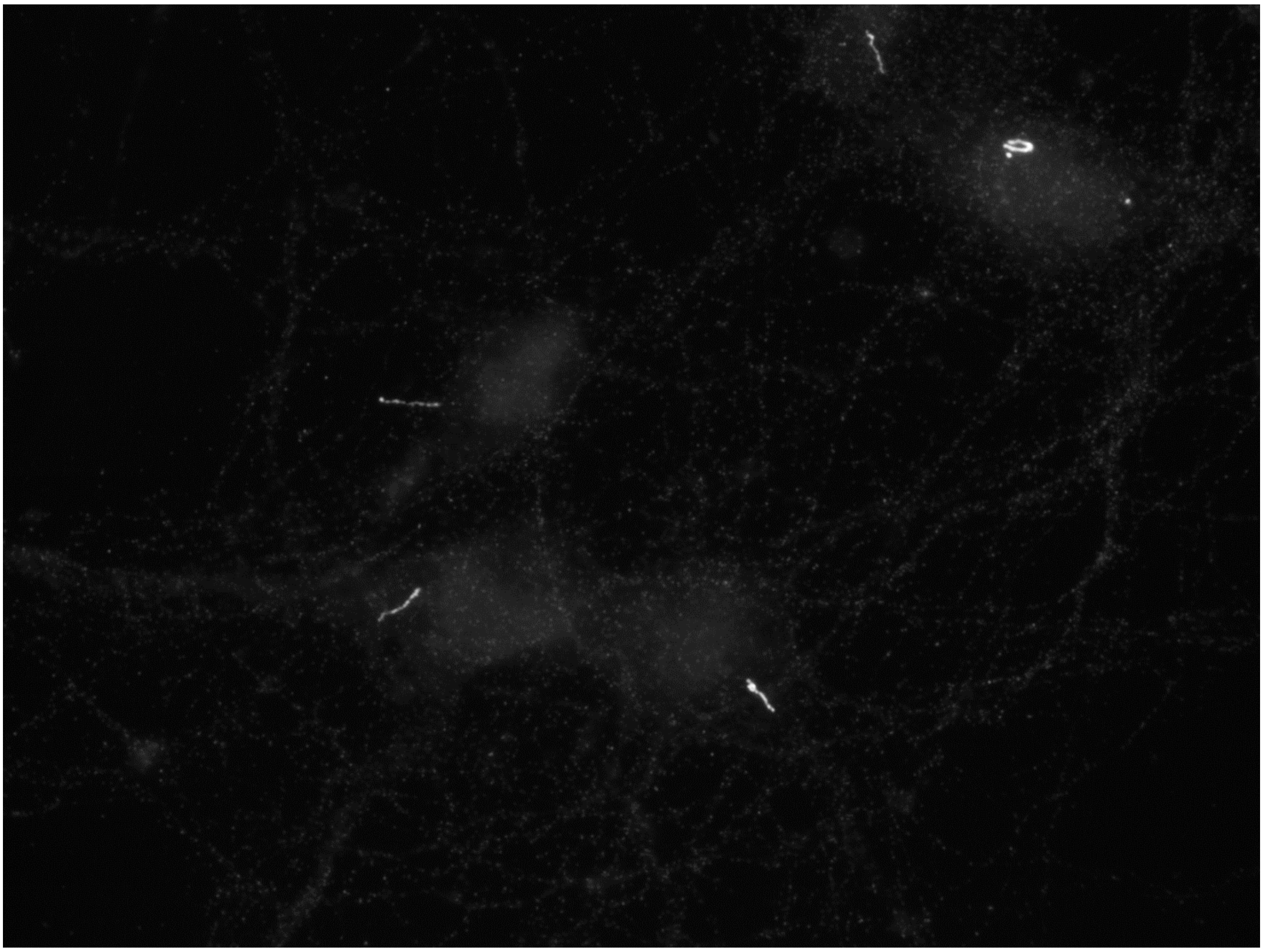

Mecp2 KO at DIV7

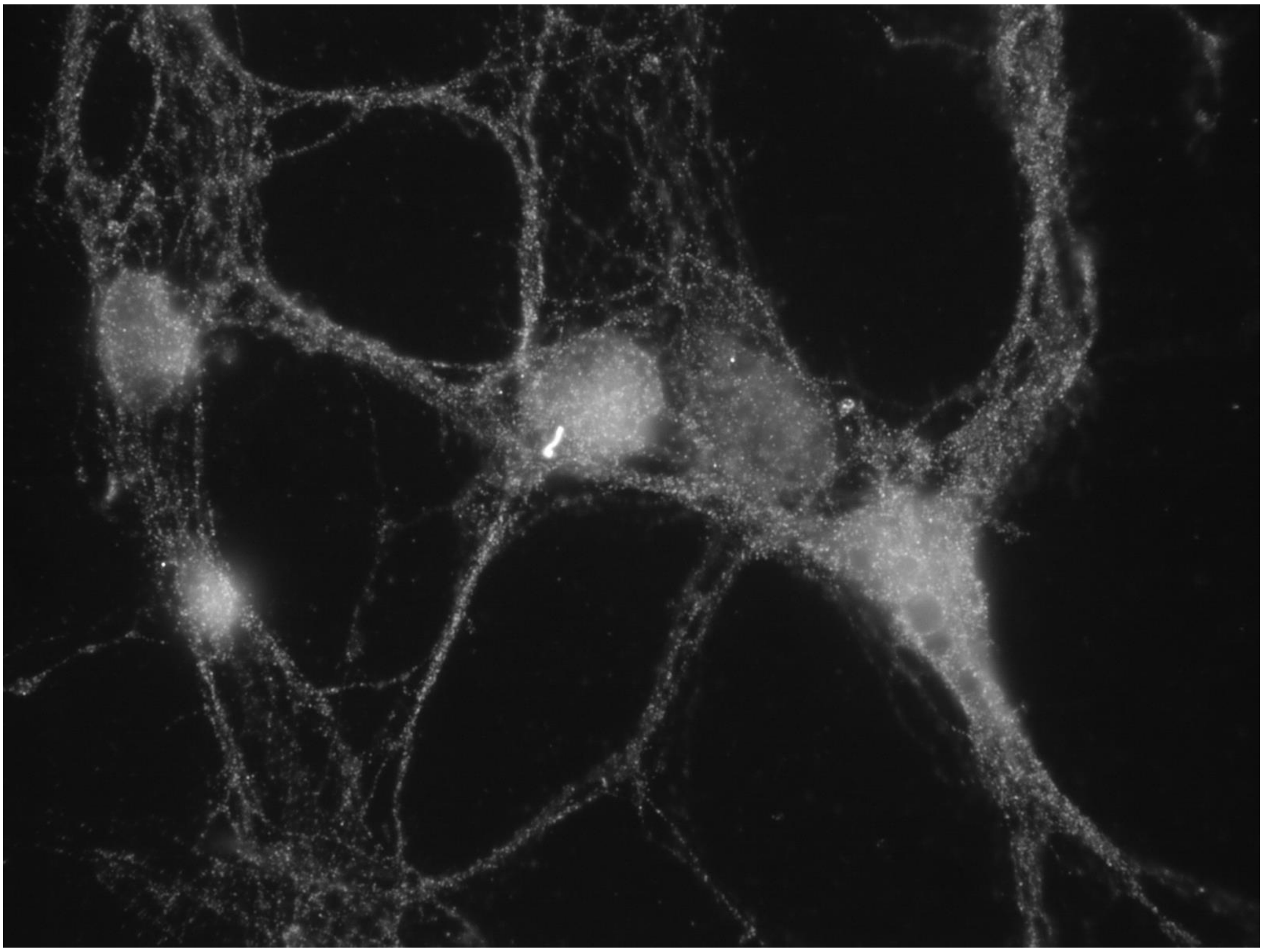

WT at DIV14

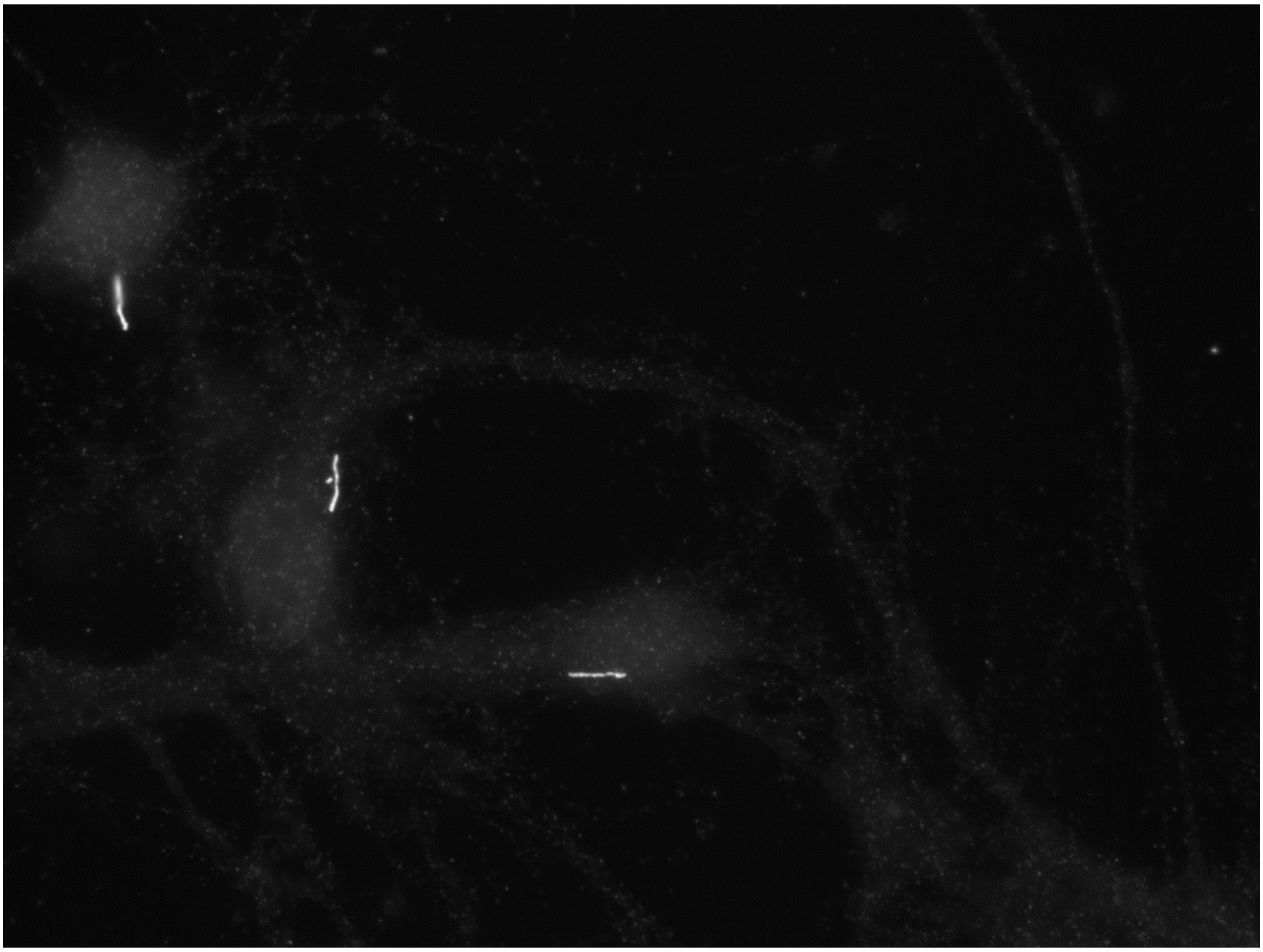

Mecp2 KO at DIV14

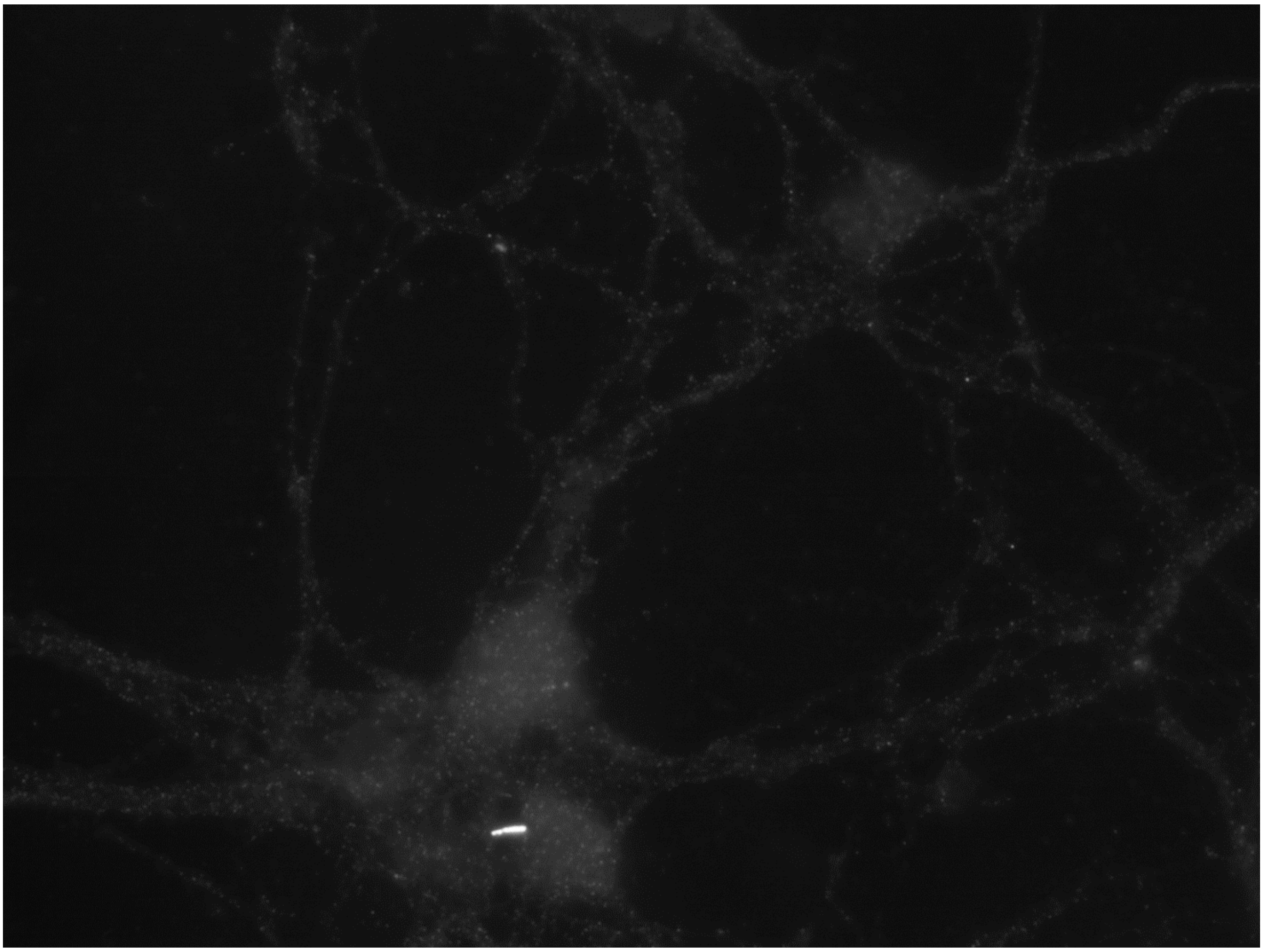

Figure 1L

WT astrocytes

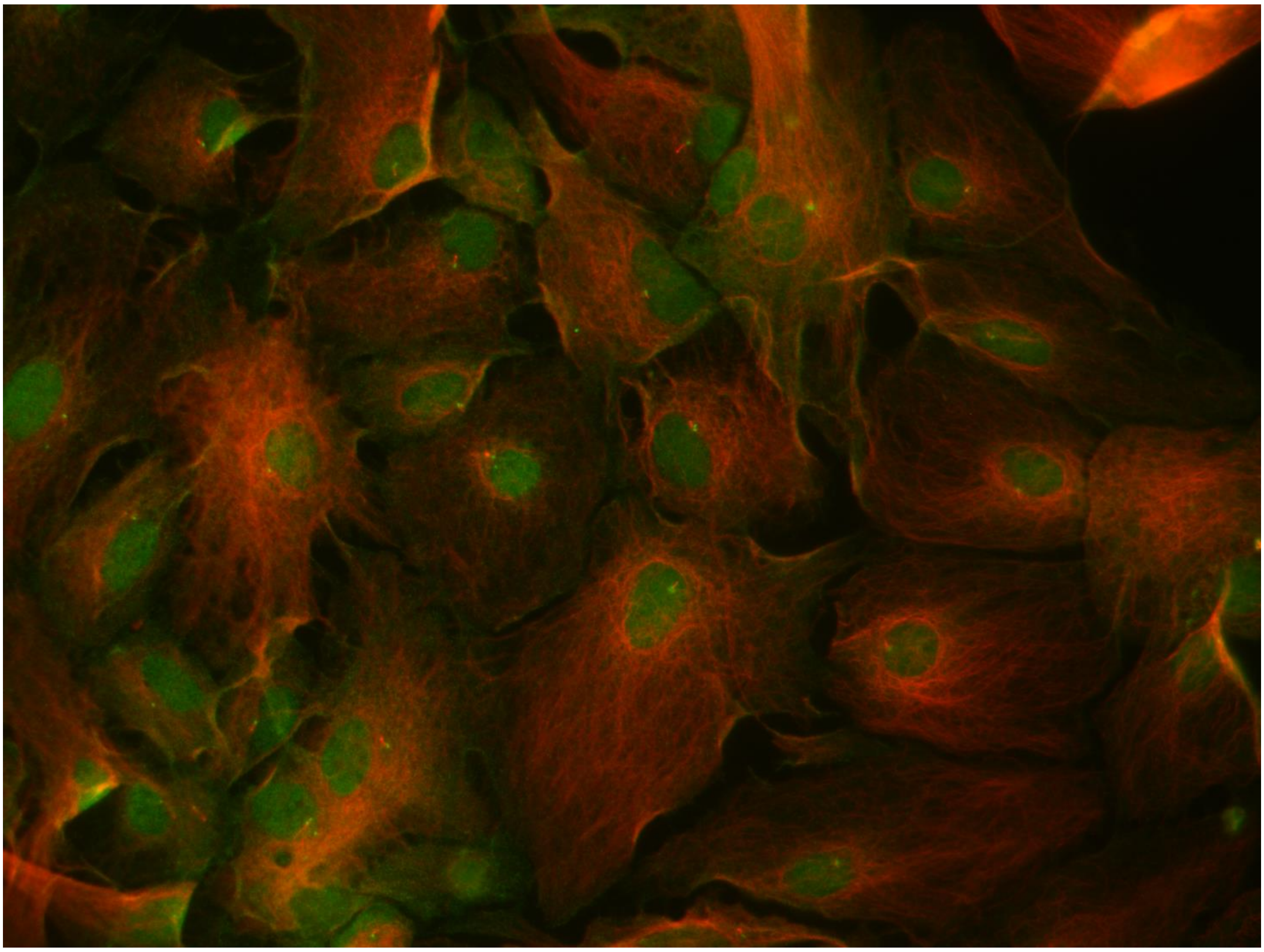

Mecp2 KO astrocytes

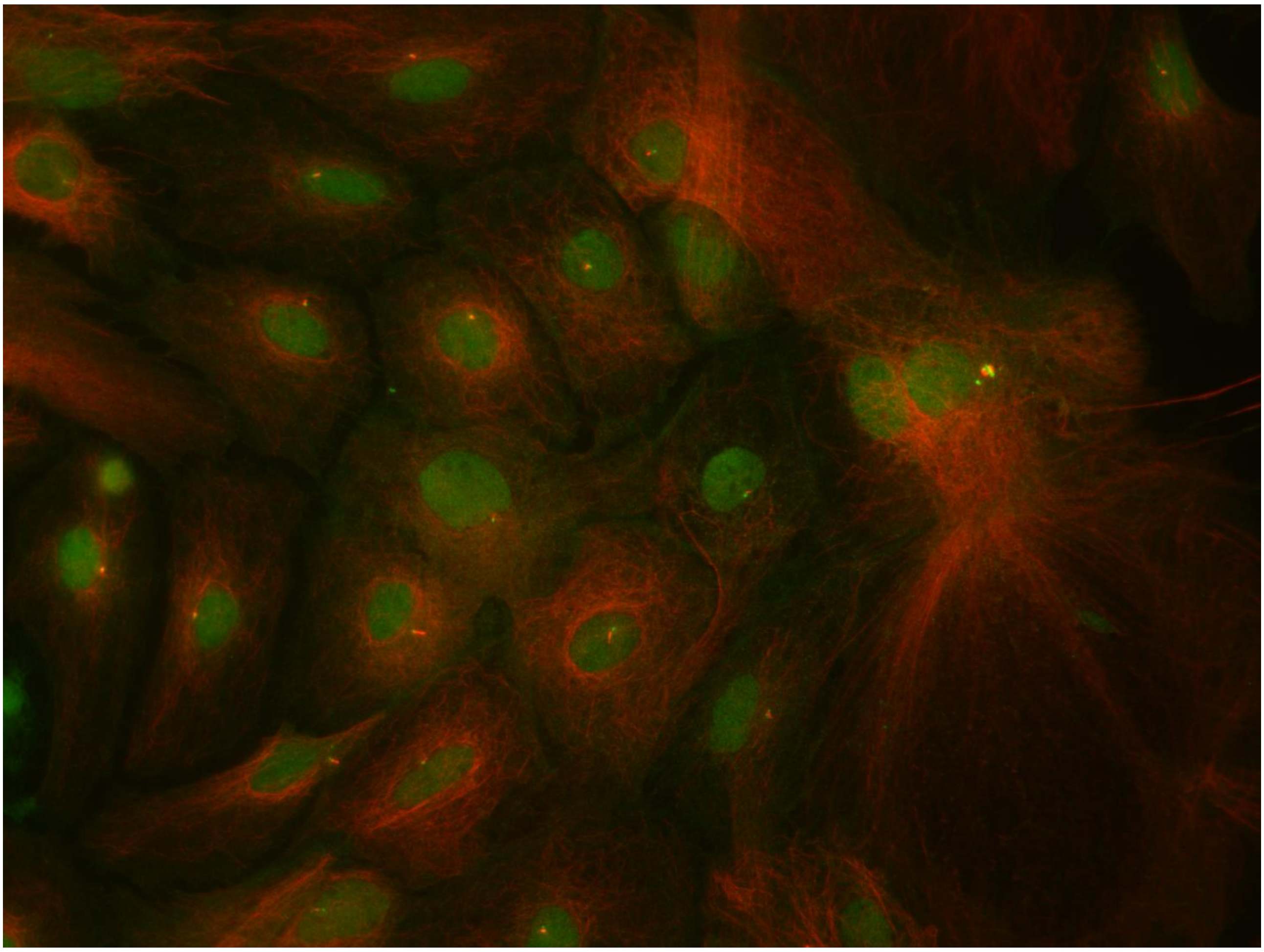

Supplement: Supplementary file 4 — Source Data for Figure 1 [file EMMM-12-e10270-s003.pdf]
