# Supplementary material for: MECP2 mutations affect ciliogenesis: a novel perspective for Rett syndrome and related disorders
Source: EMBO Mol Med. 2020 May 8;12(6):e10270. doi: 10.15252/emmm.201910270 (PMC7278541; doi:10.15252/emmm.201910270)

Figure 2A

Immunofluorescence on Mecp2 null neurons infected with a GFP expressing lentivirus

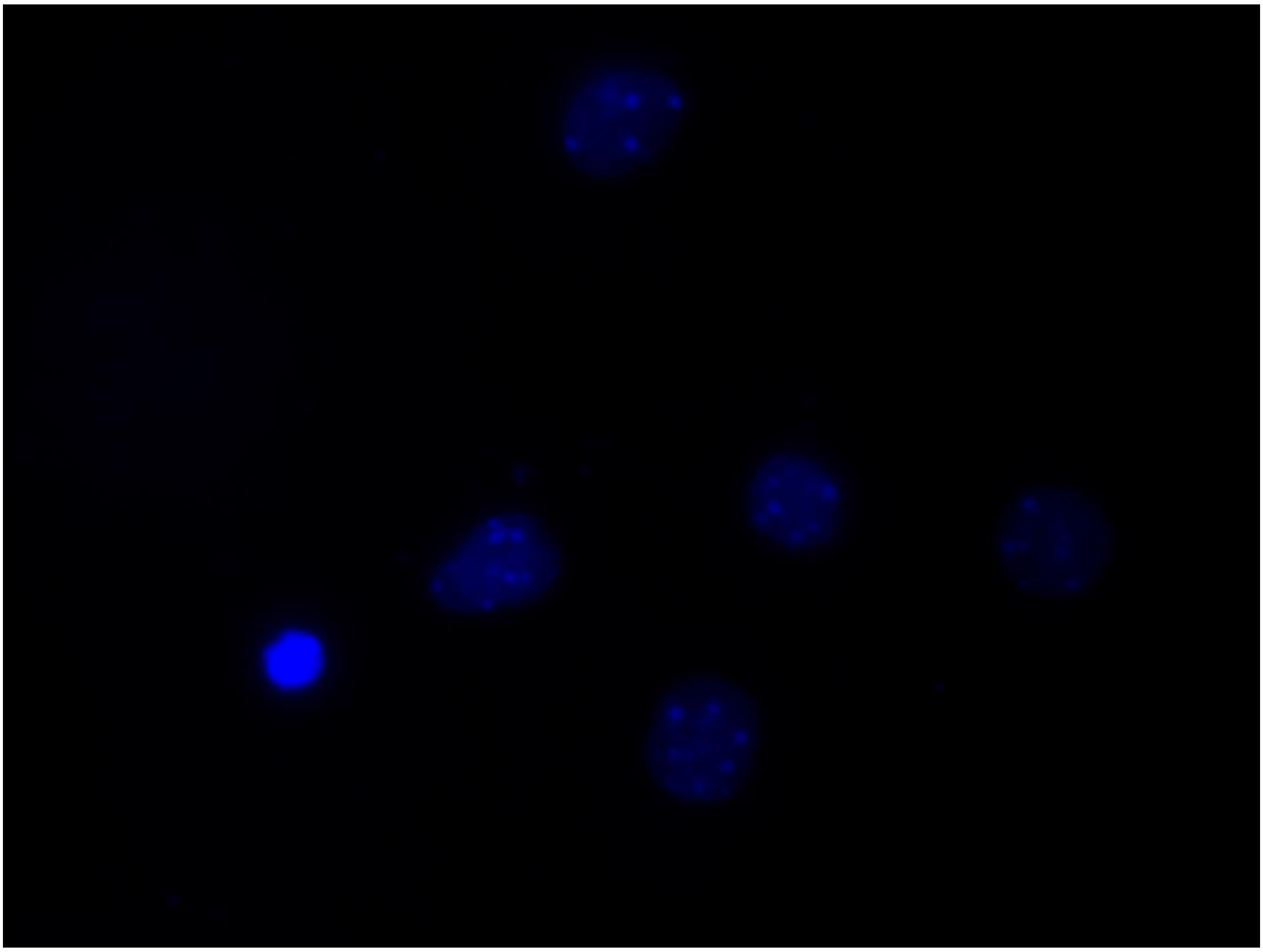

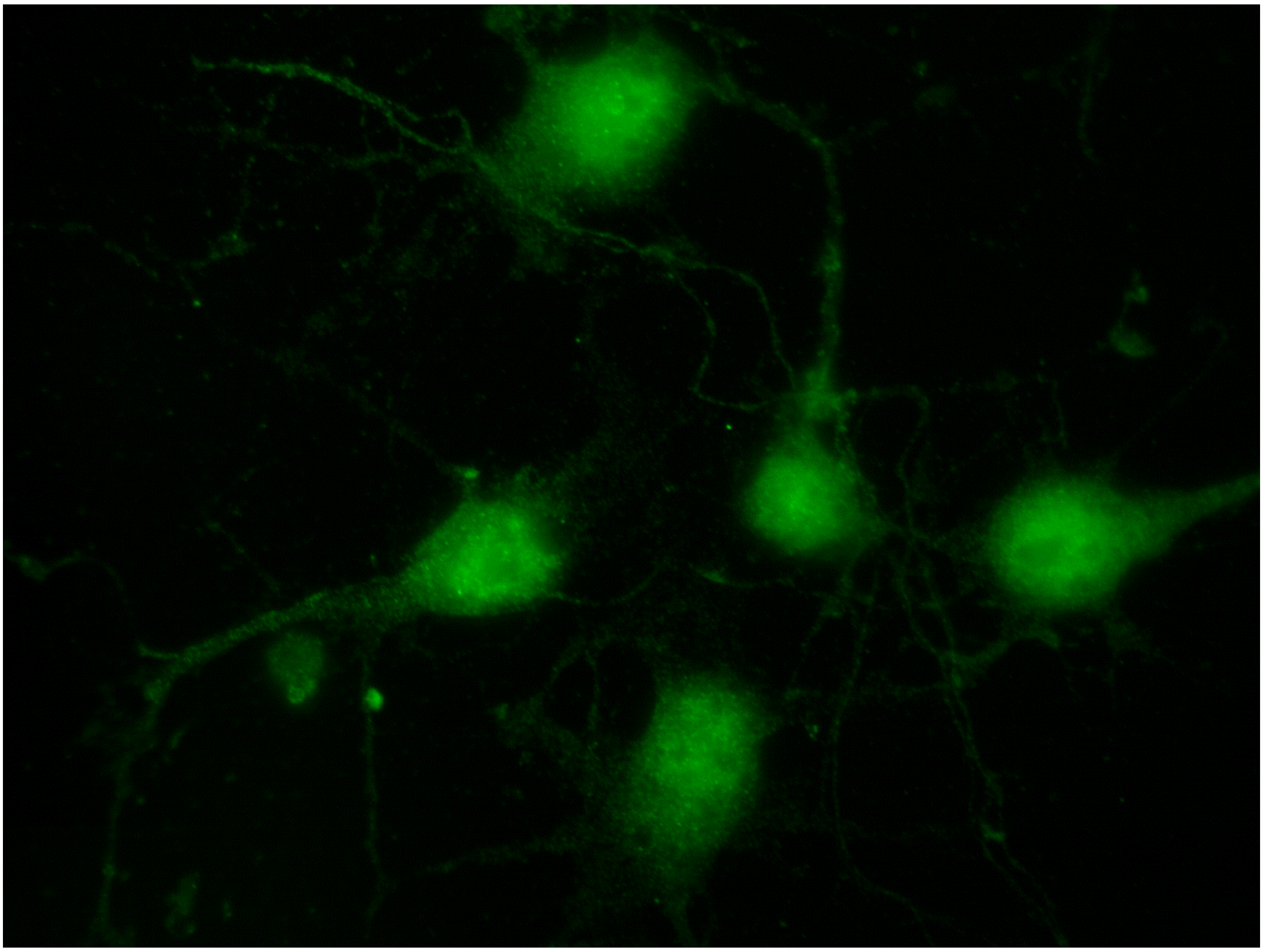

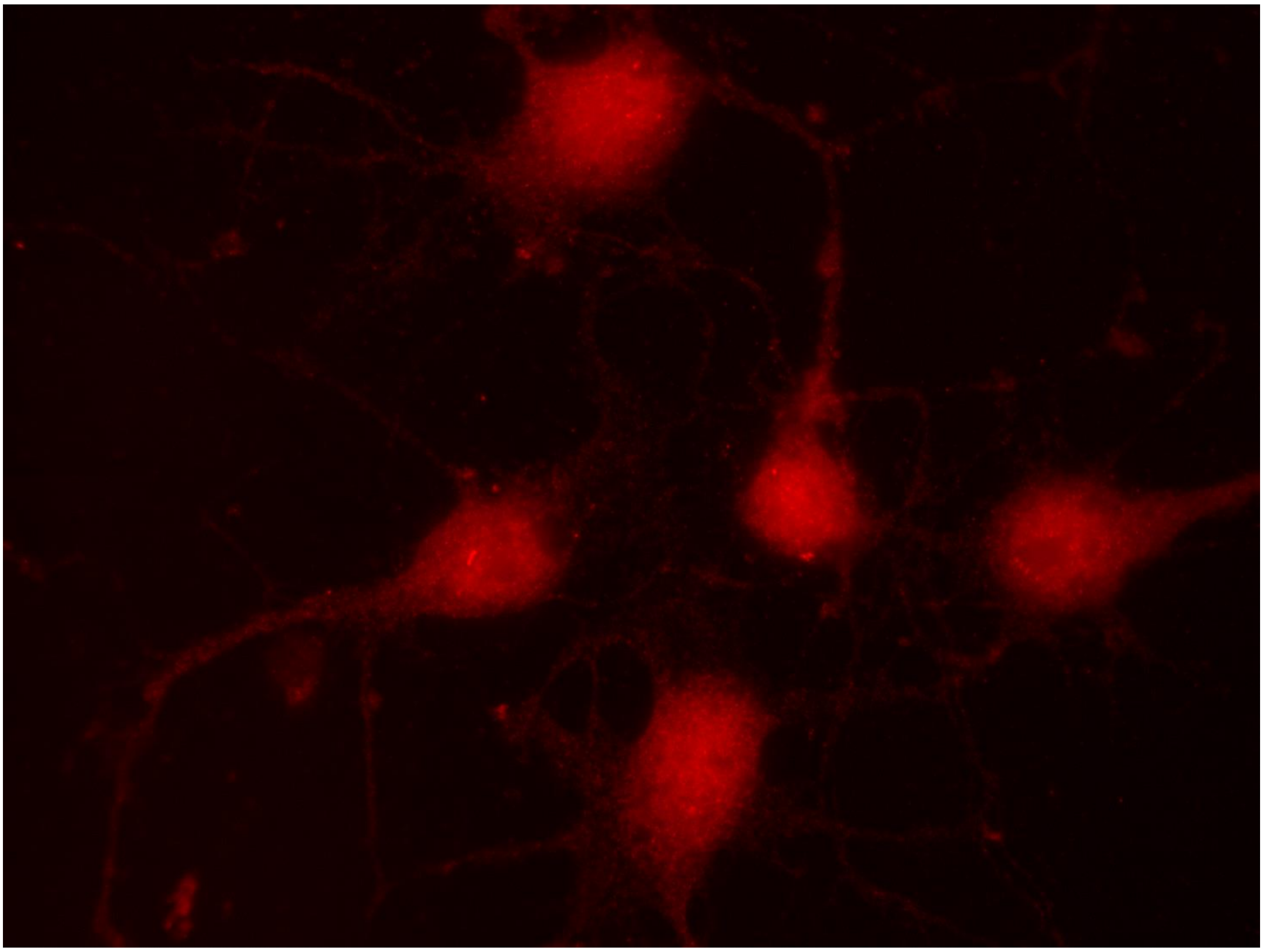

Immunofluorescence on Mecp2 null neurons infected with MeCP2-GFP expressing lentivirus

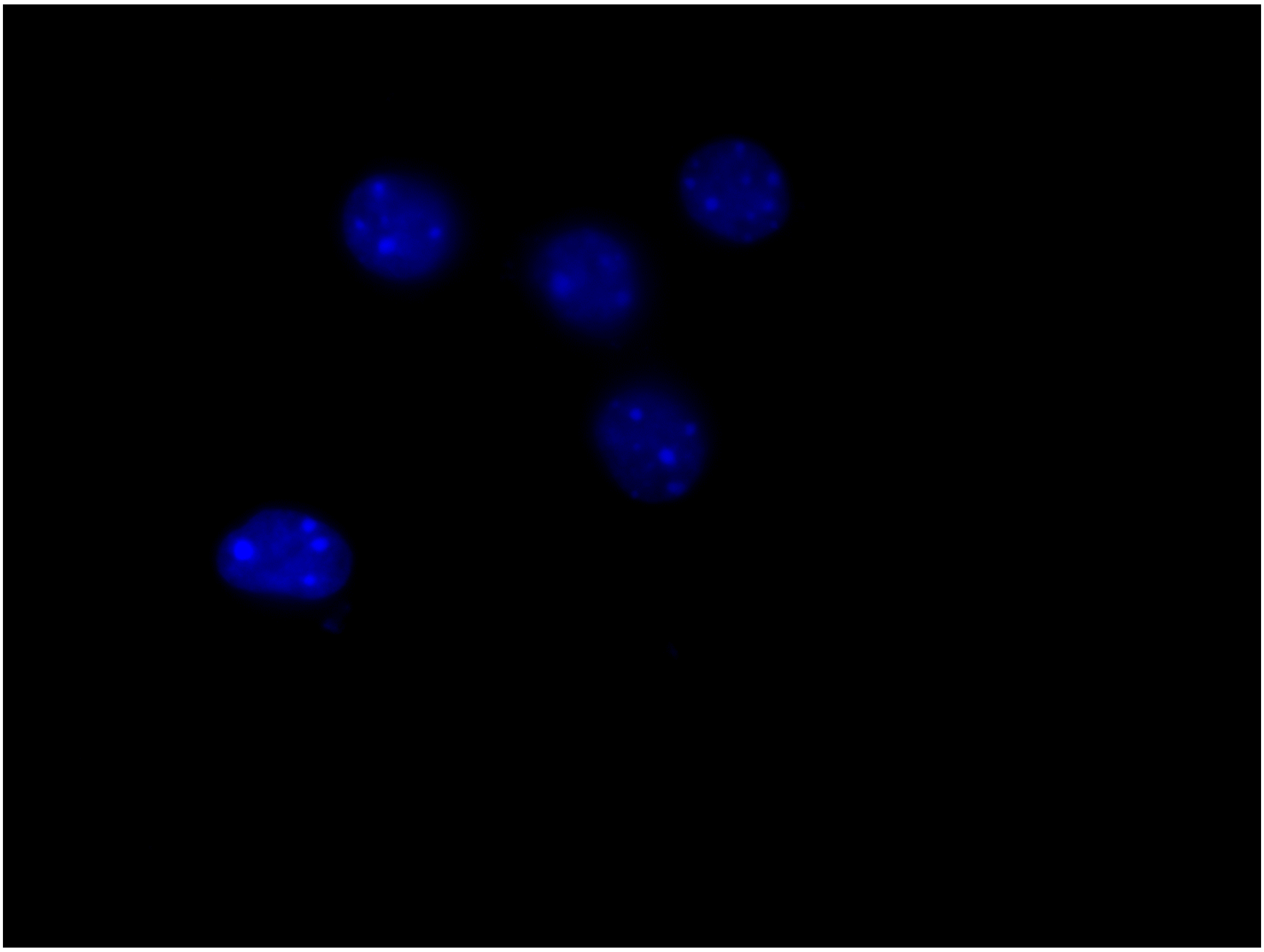

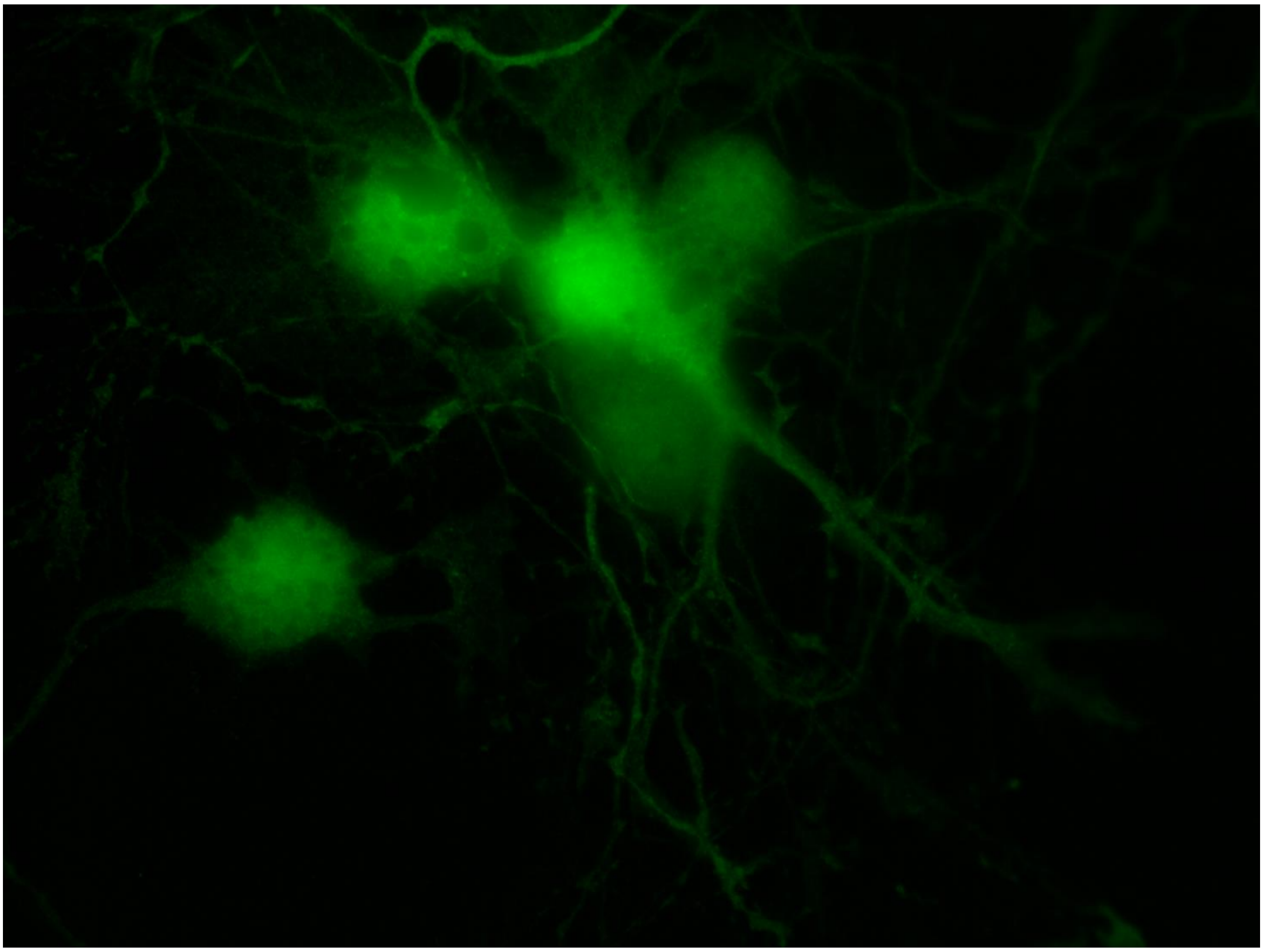

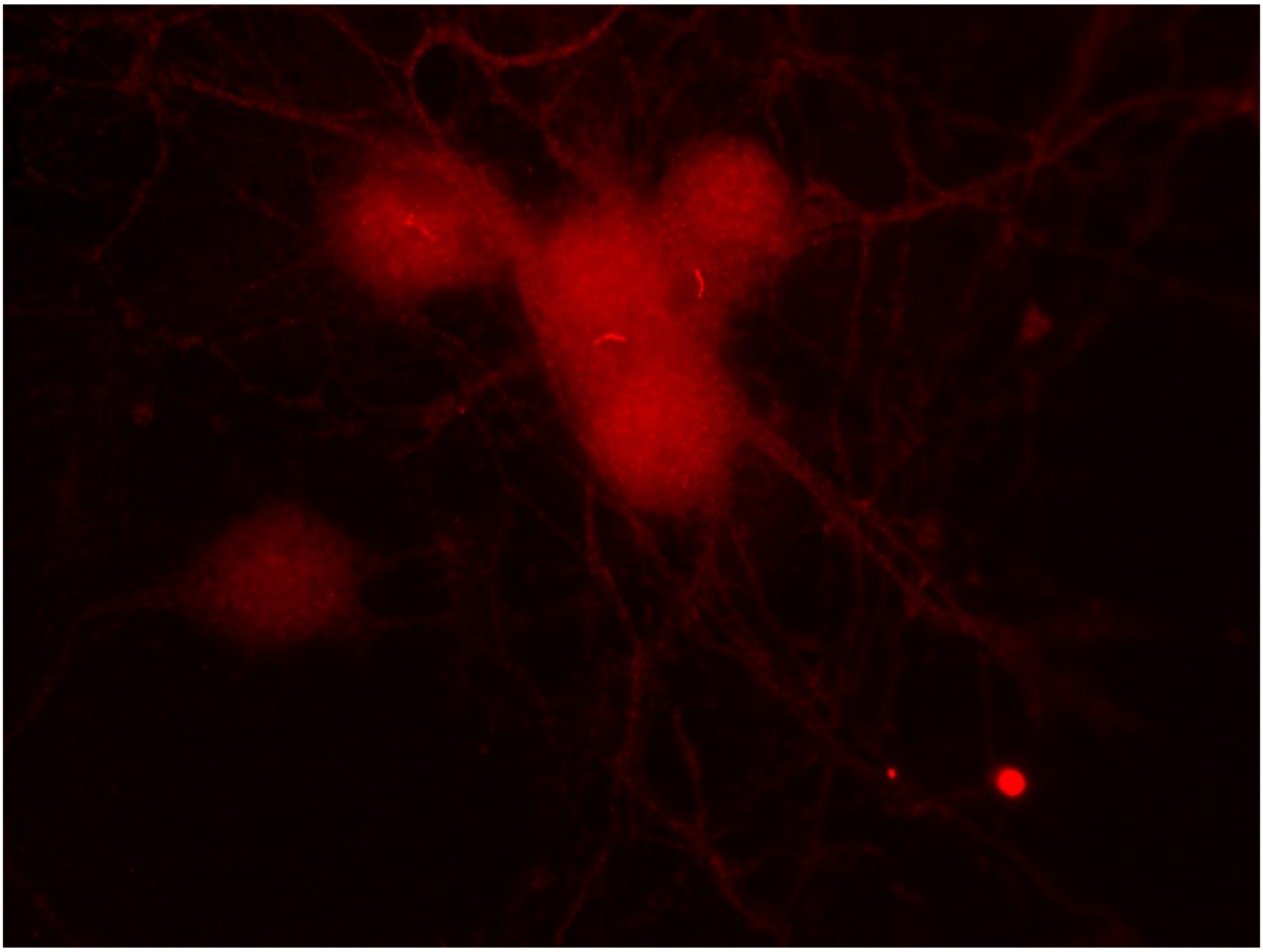

Figure 2B. WB for Mecp2 on neuronal proteins

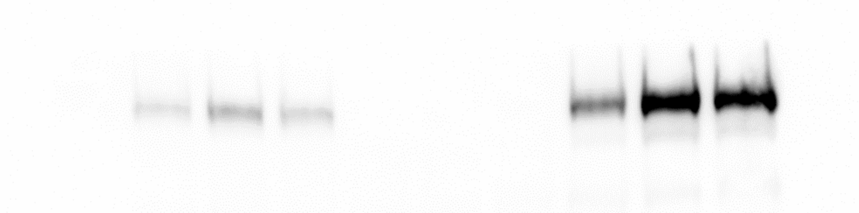

Marker

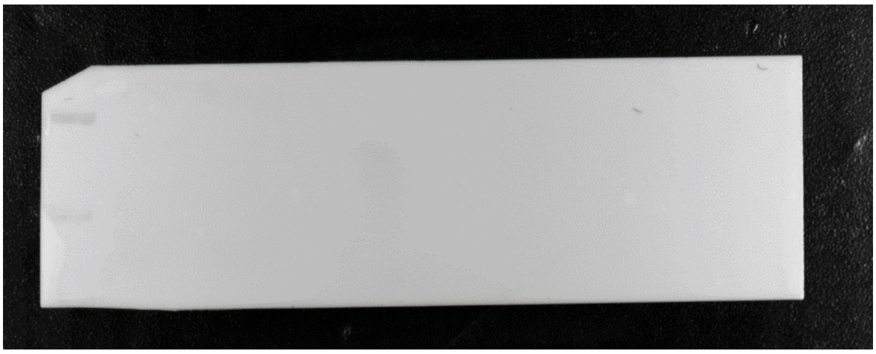

TGX-stain free

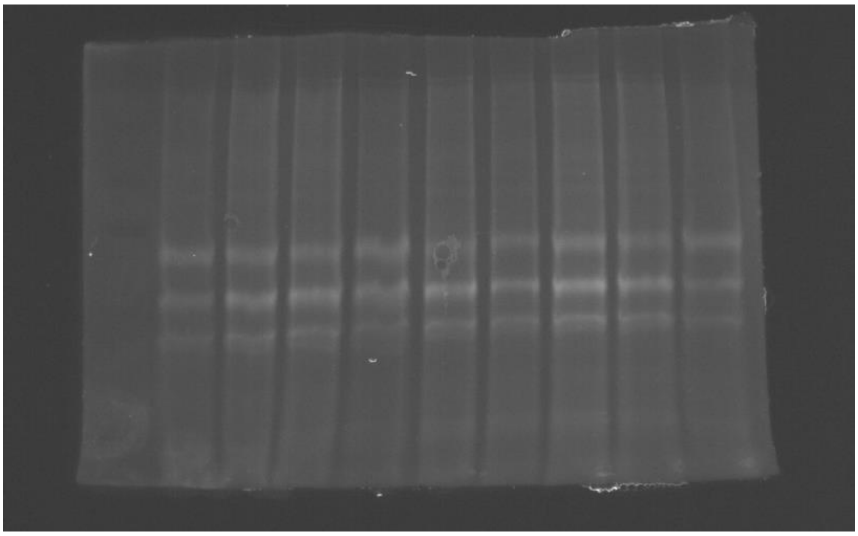

Supplement: Supplementary file 5 — Source Data for Figure 2 [file EMMM-12-e10270-s004.pdf]
