# Supplementary material for: MECP2 mutations affect ciliogenesis: a novel perspective for Rett syndrome and related disorders
Source: EMBO Mol Med. 2020 May 8;12(6):e10270. doi: 10.15252/emmm.201910270 (PMC7278541; doi:10.15252/emmm.201910270)

Figure 3D

WB for Gli1 and marker

Marker

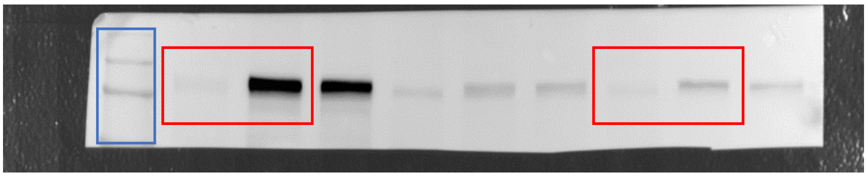

WB for Mecp2 and marker

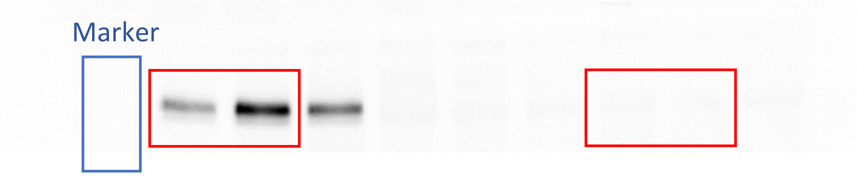

Marker

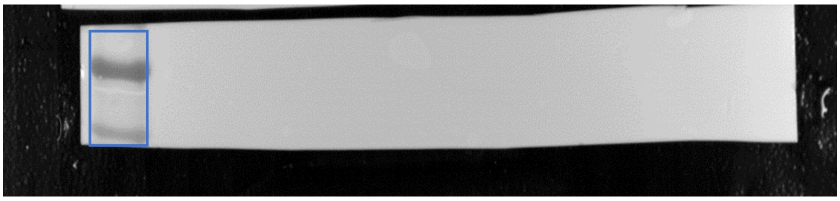

WB for α-tubulin and marker

Marker

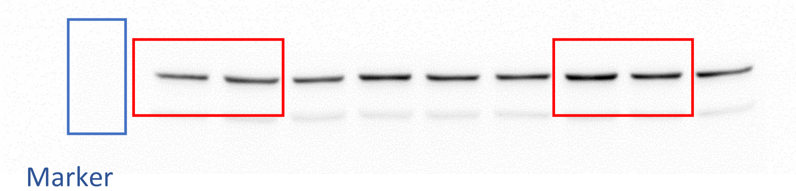

Marker

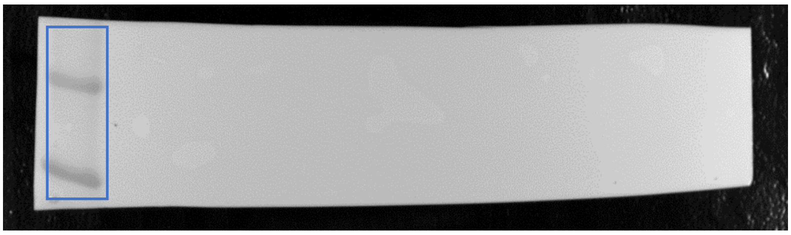

Figure 3E

WB for Gli1 and marker

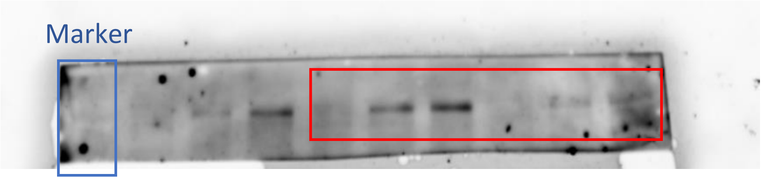

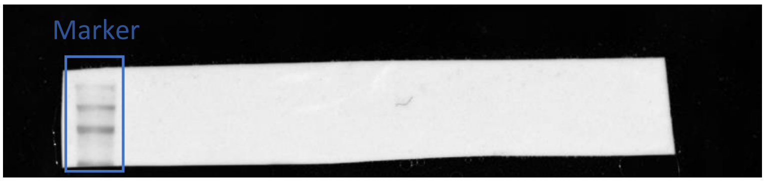

WB for α-tubulin

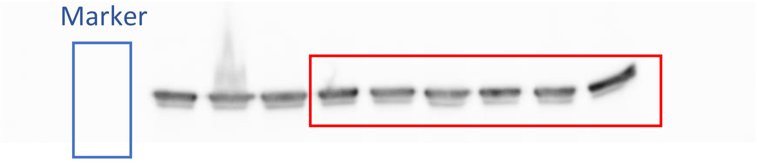

WB for Mecp2 and marker

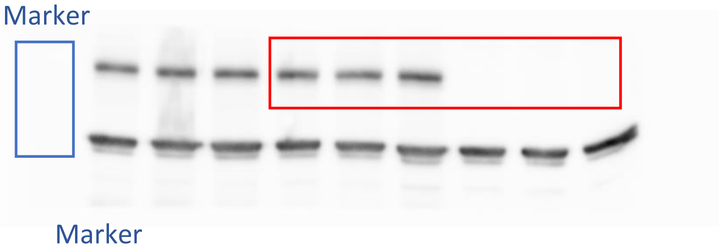

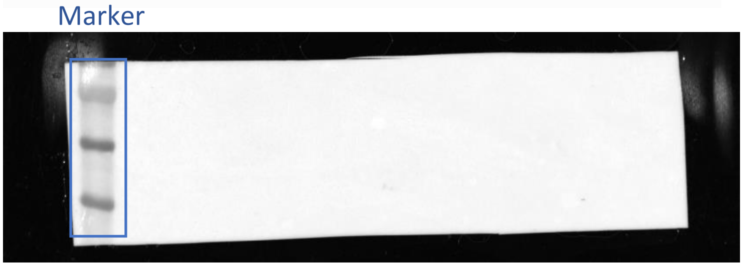

Supplement: Supplementary file 6 — Source Data for Figure 3 [file EMMM-12-e10270-s005.pdf]
