# Supplementary material for: MECP2 mutations affect ciliogenesis: a novel perspective for Rett syndrome and related disorders
Source: EMBO Mol Med. 2020 May 8;12(6):e10270. doi: 10.15252/emmm.201910270 (PMC7278541; doi:10.15252/emmm.201910270)

Figure 4A

WT

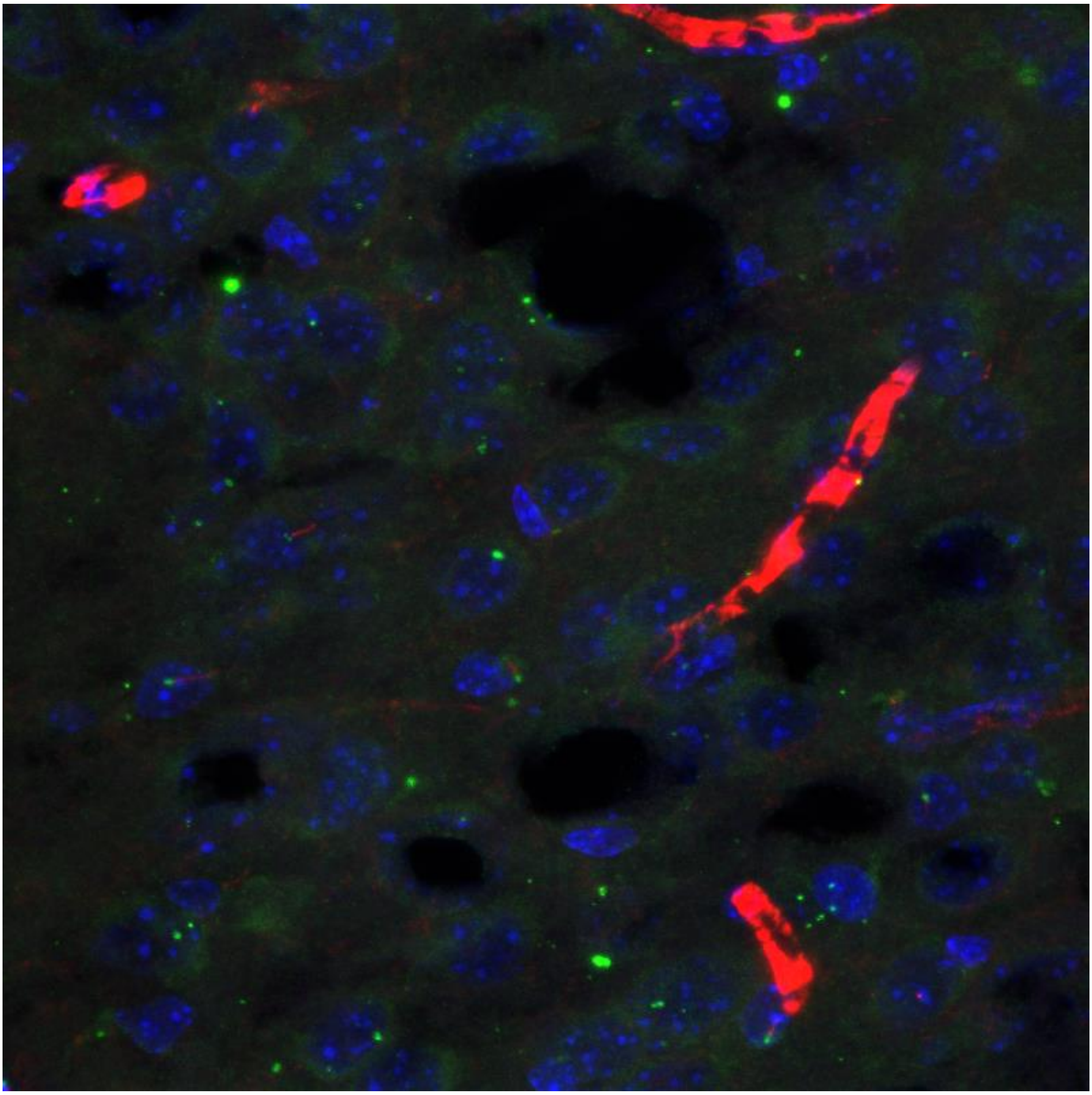

Mecp2 null

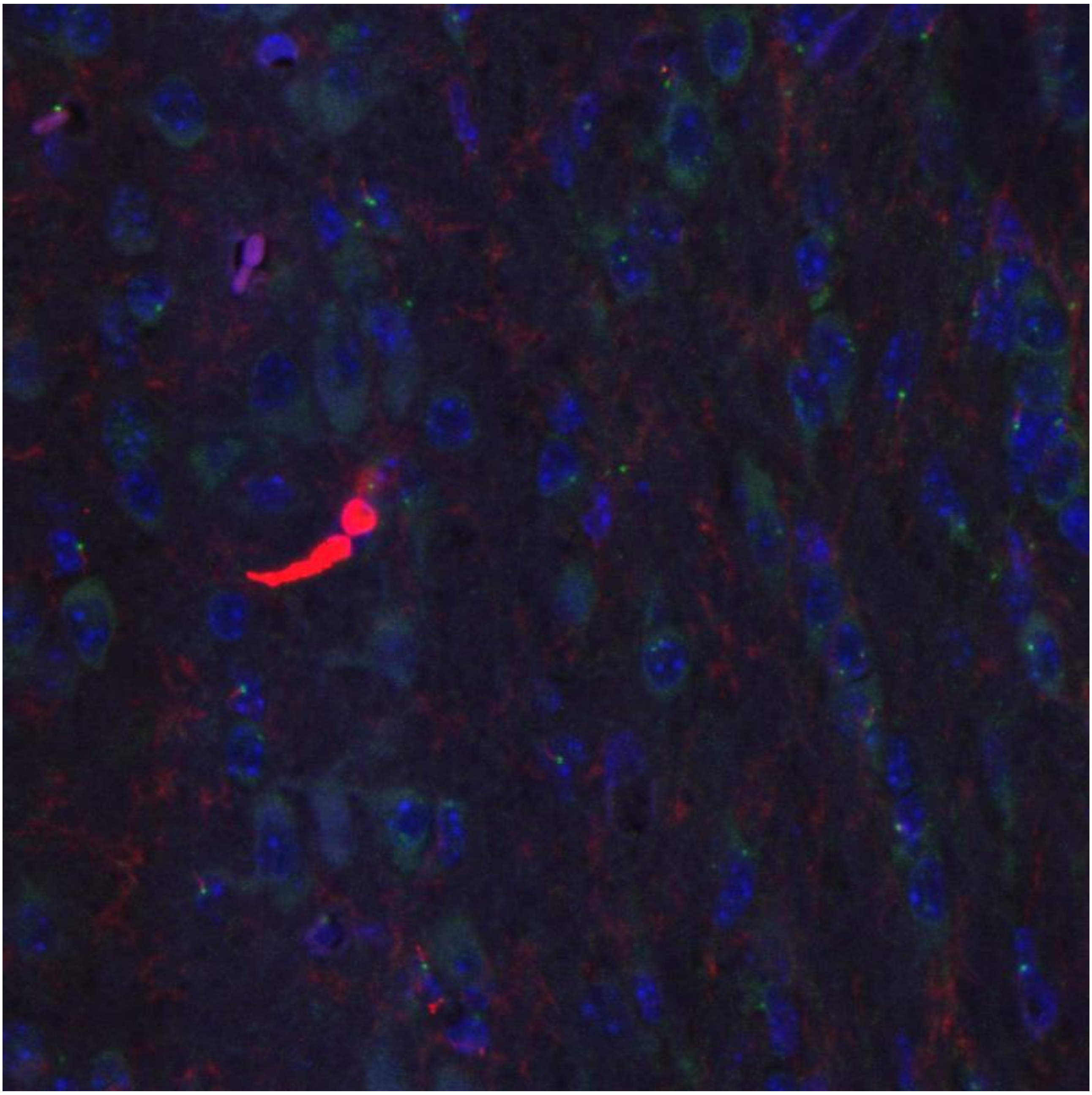

Figure 4D

WT IGL

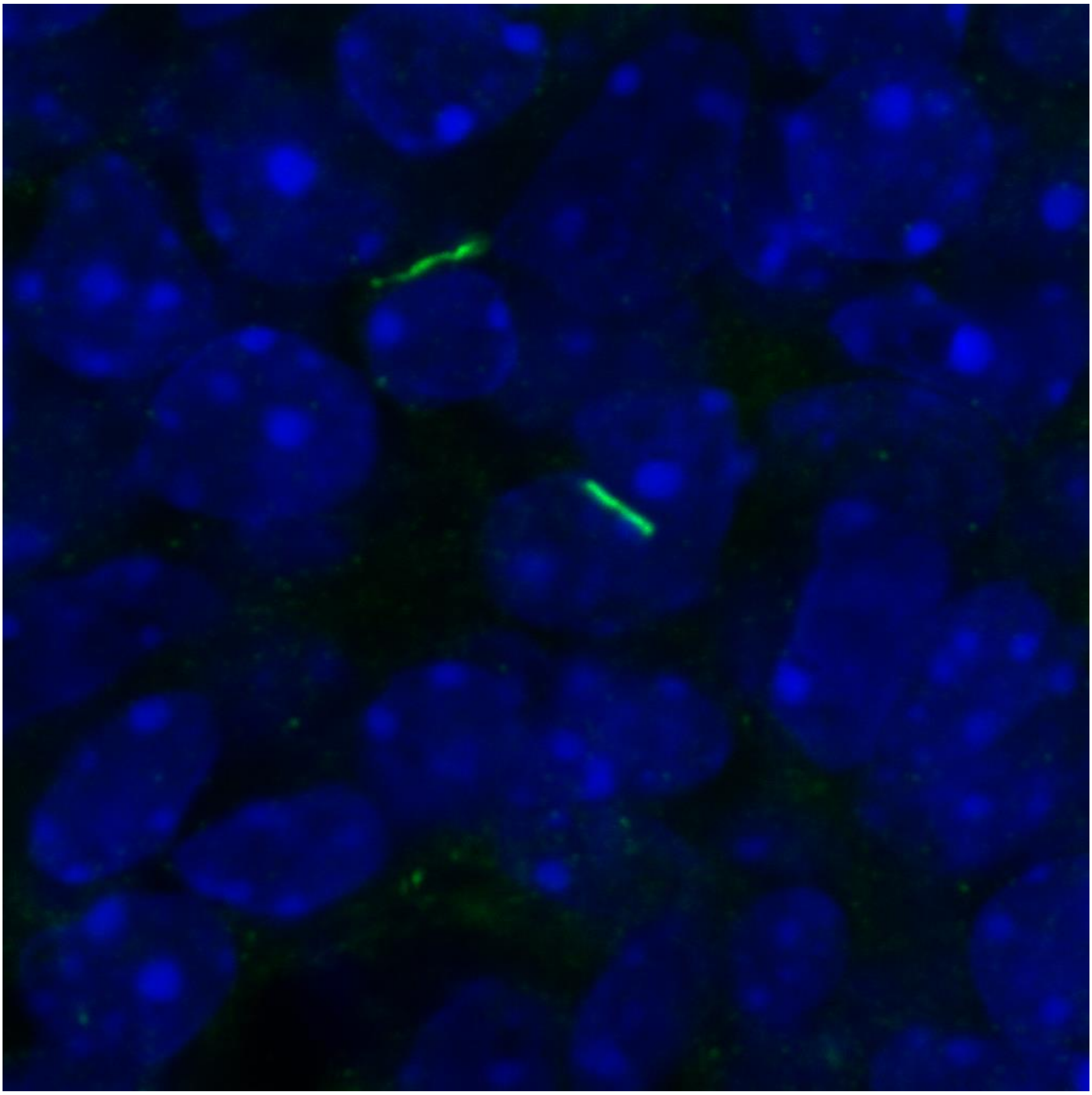

Mecp2 null IGL

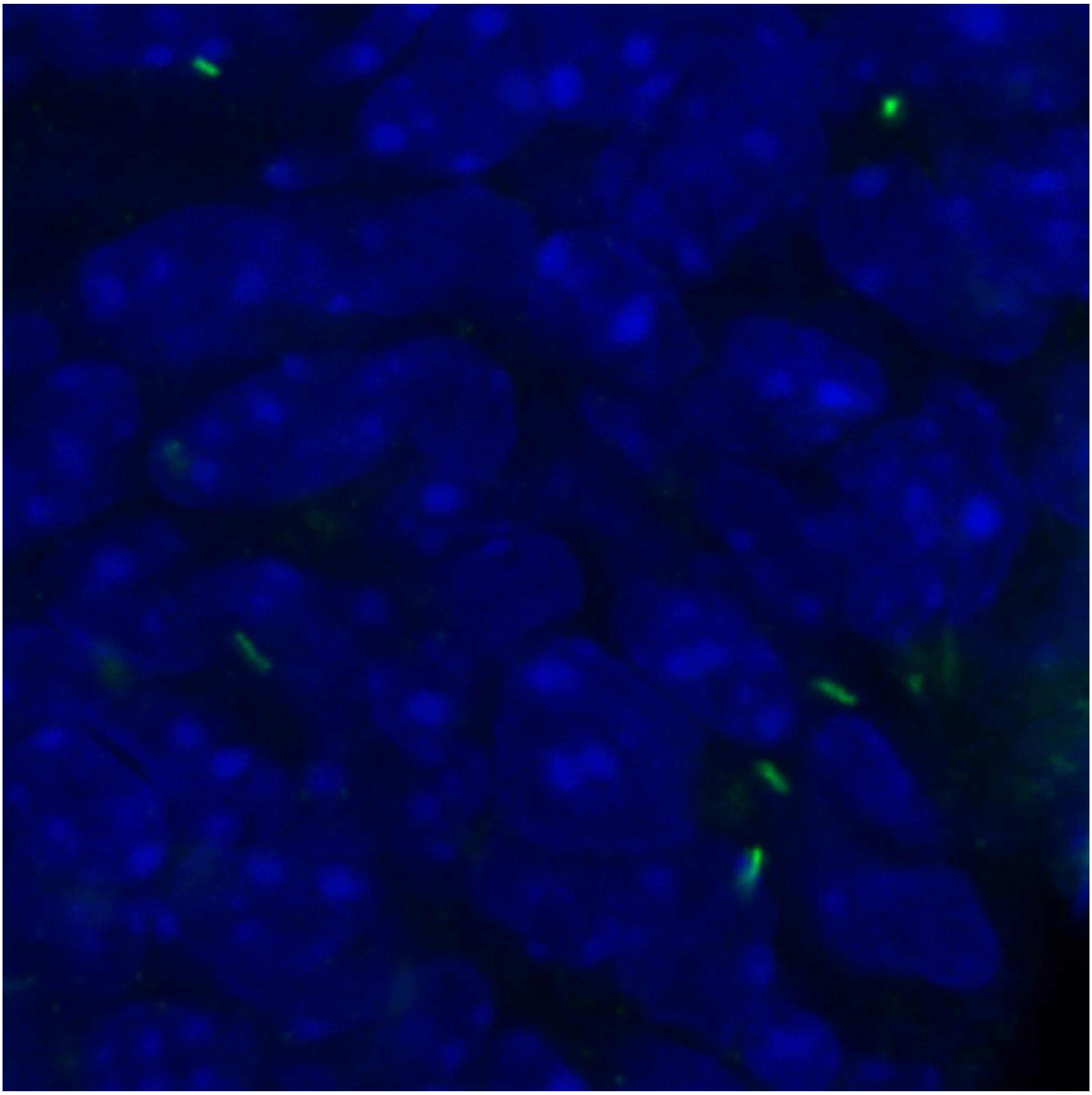

Mecp2 Het IGL

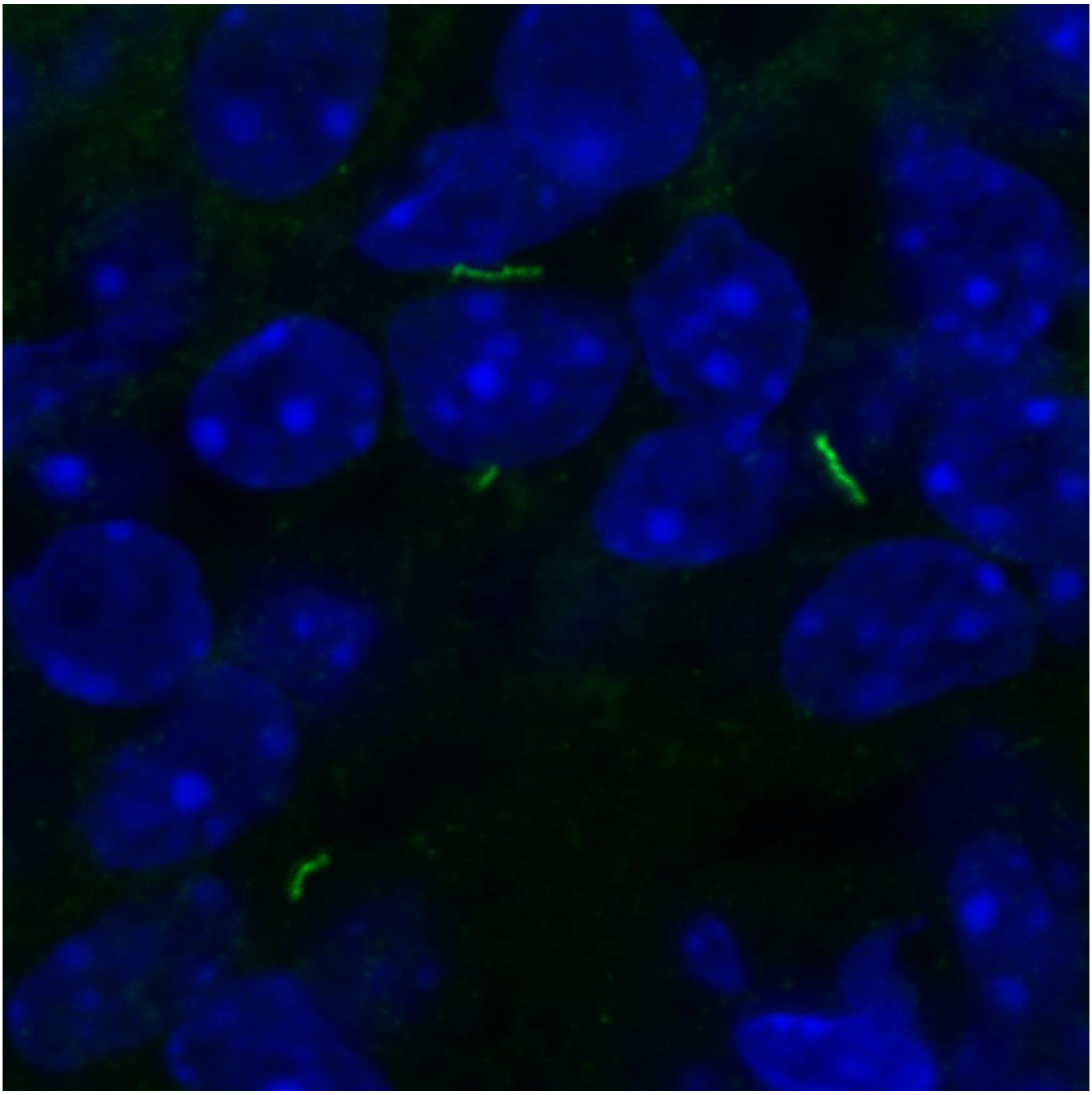

WT EGL

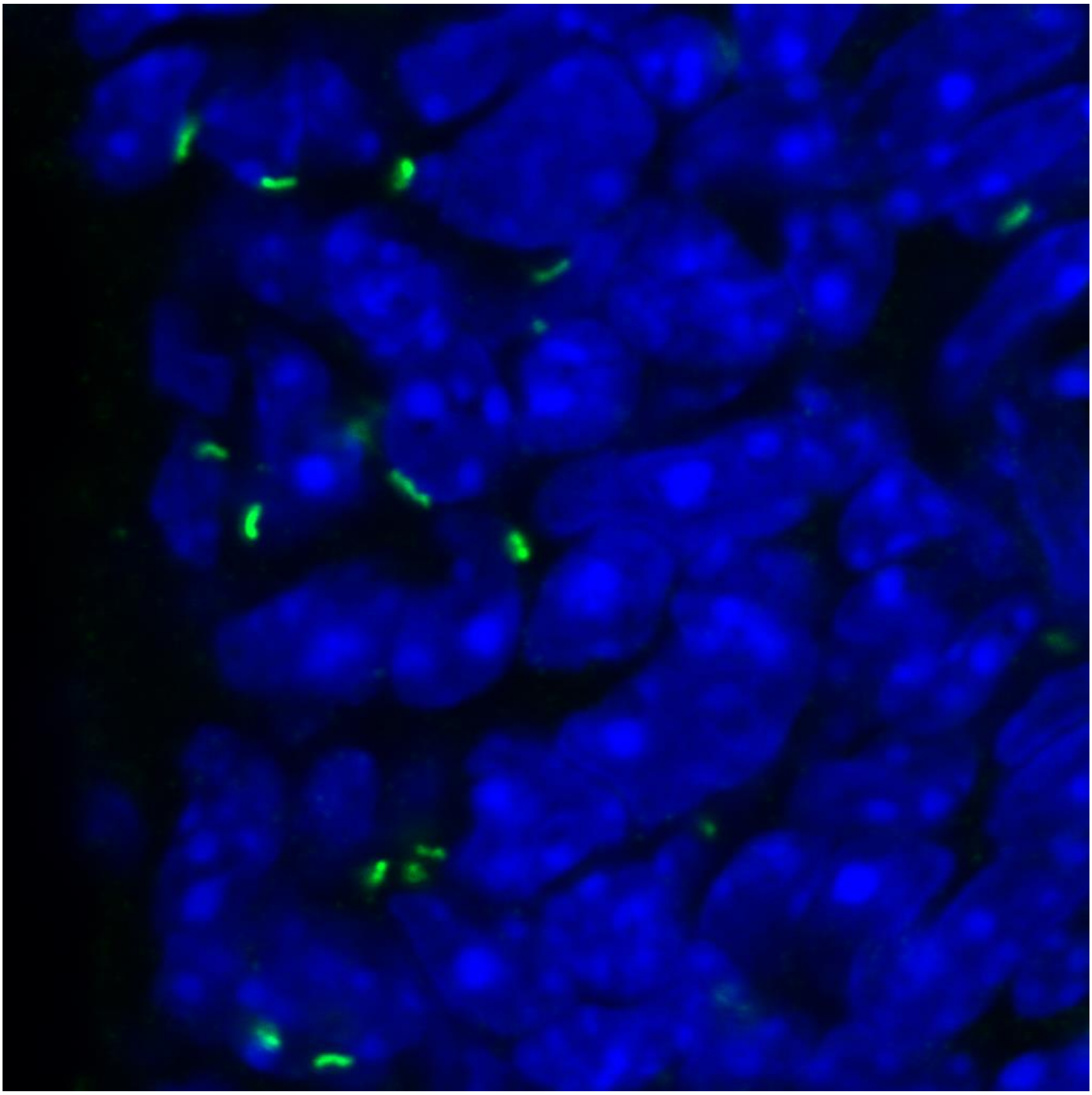

Mecp2 null EGL

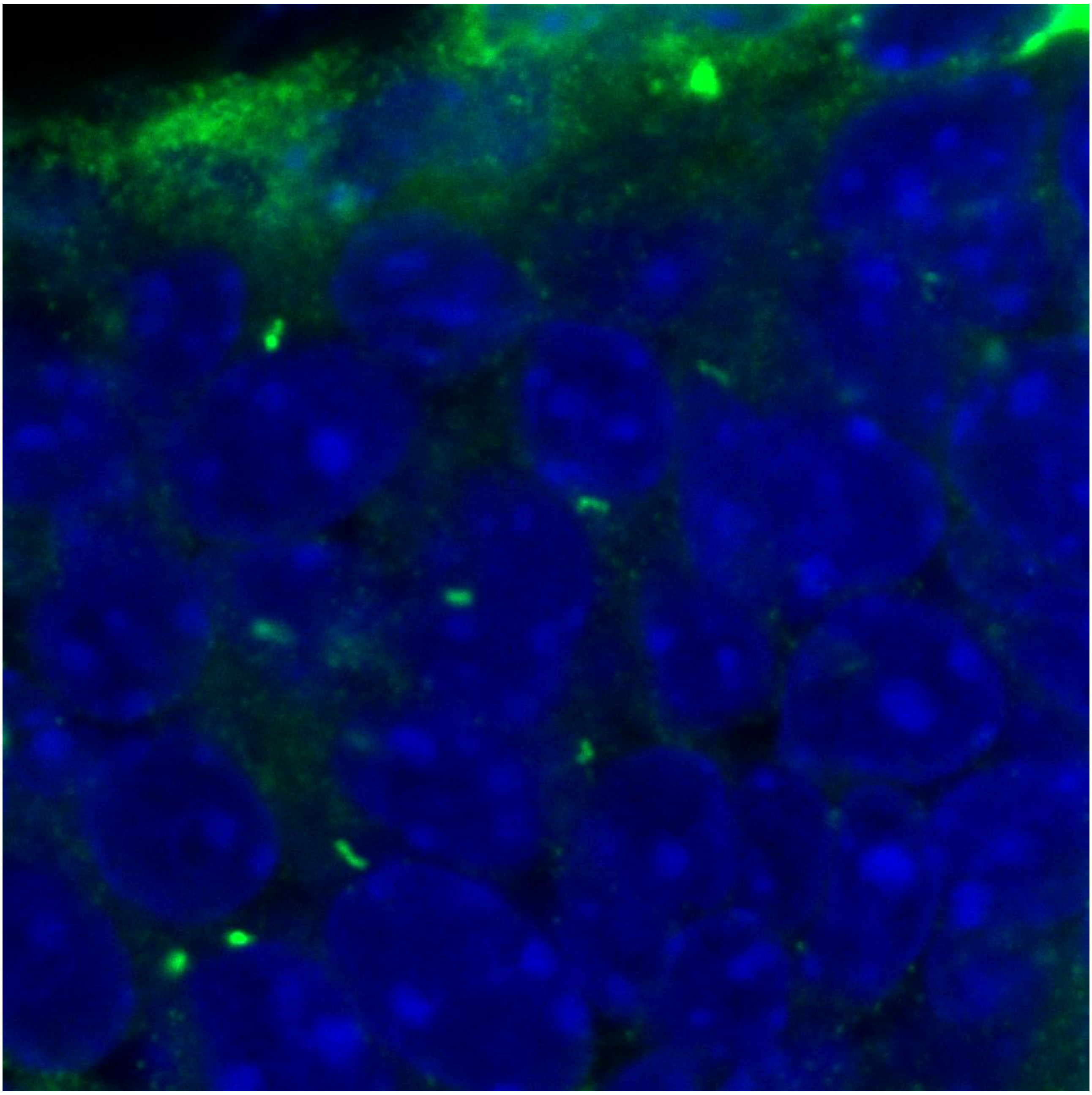

Mecp2 Het EGL

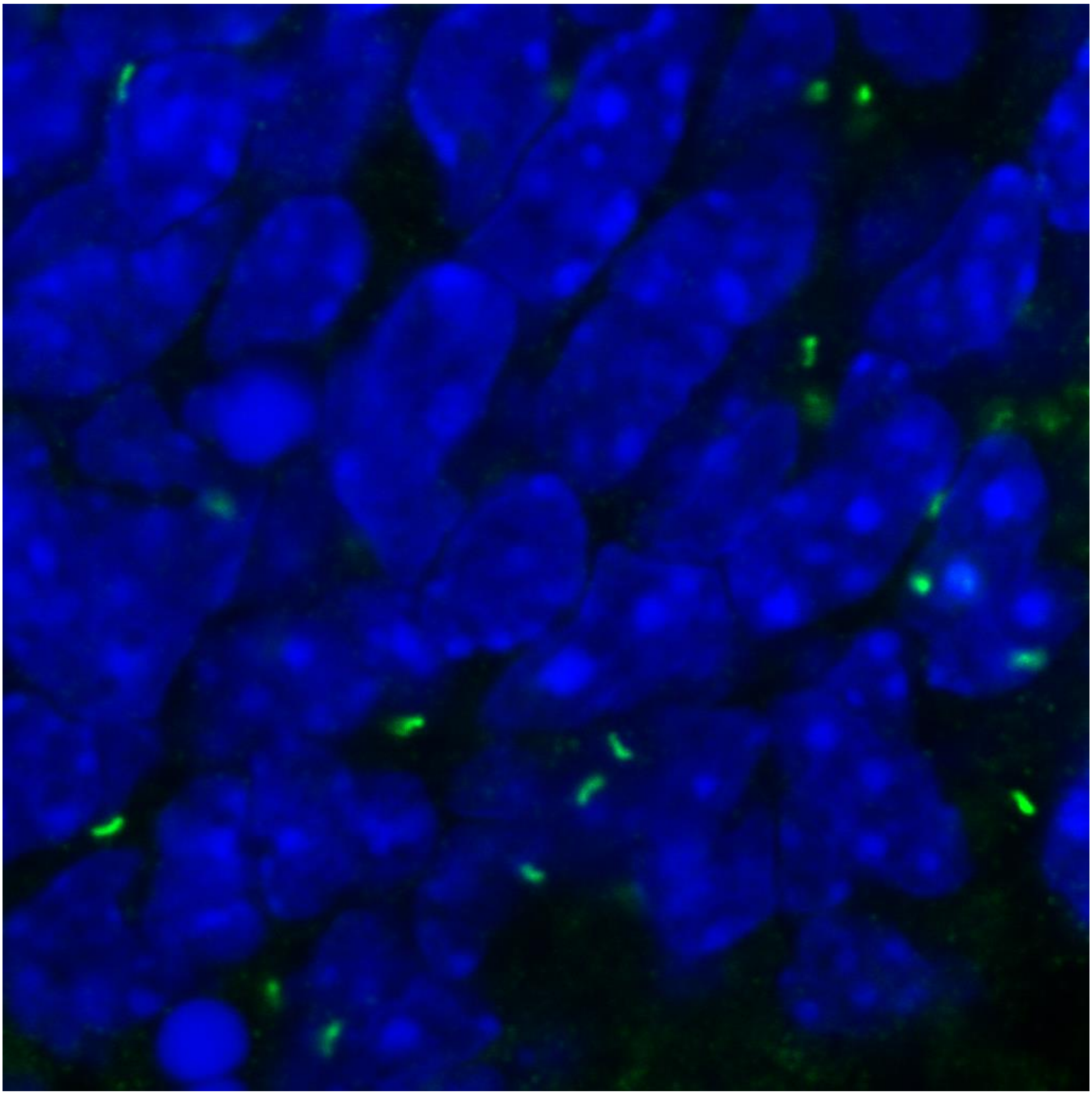

Figure 4G

WB for Gli1

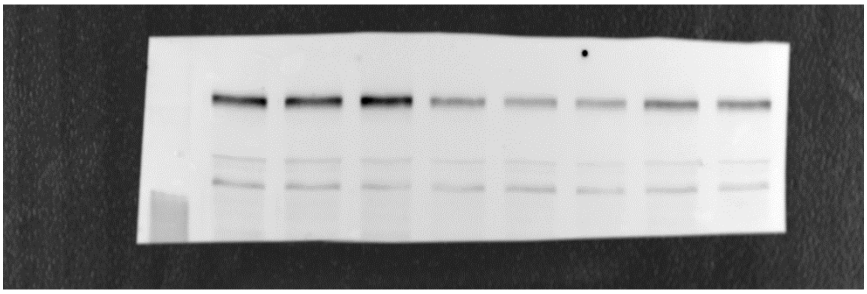

WB for α-tubulin

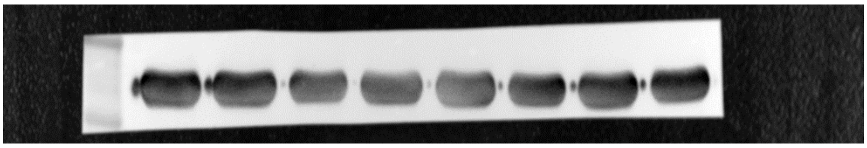

Supplement: Supplementary file 7 — Source Data for Figure 4 [file EMMM-12-e10270-s006.pdf]
