# Supplementary material for: MECP2 mutations affect ciliogenesis: a novel perspective for Rett syndrome and related disorders
Source: EMBO Mol Med. 2020 May 8;12(6):e10270. doi: 10.15252/emmm.201910270 (PMC7278541; doi:10.15252/emmm.201910270)

Figure 5D

Ctrl not-treated

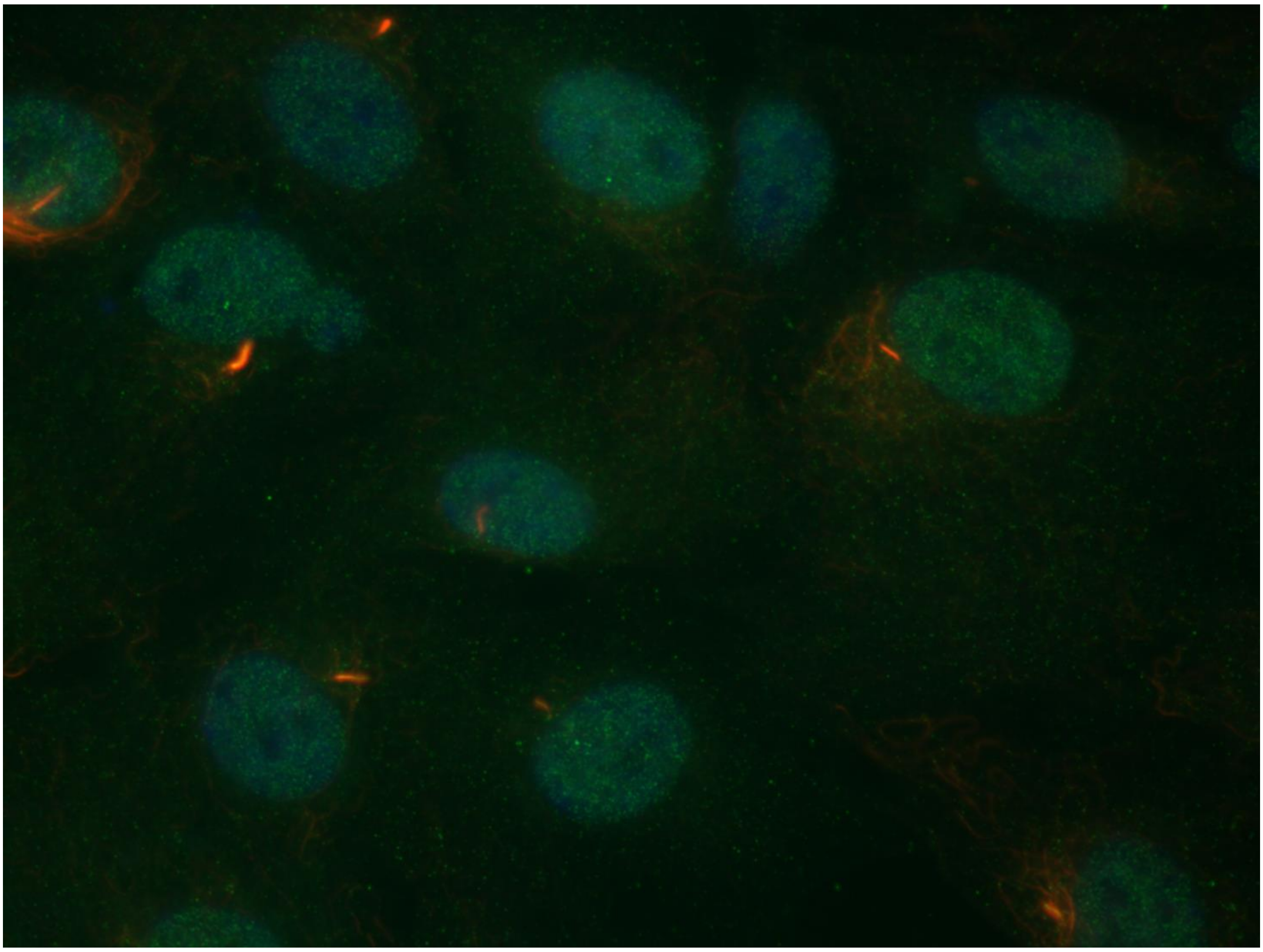

Ctrl-SAG 200 nM

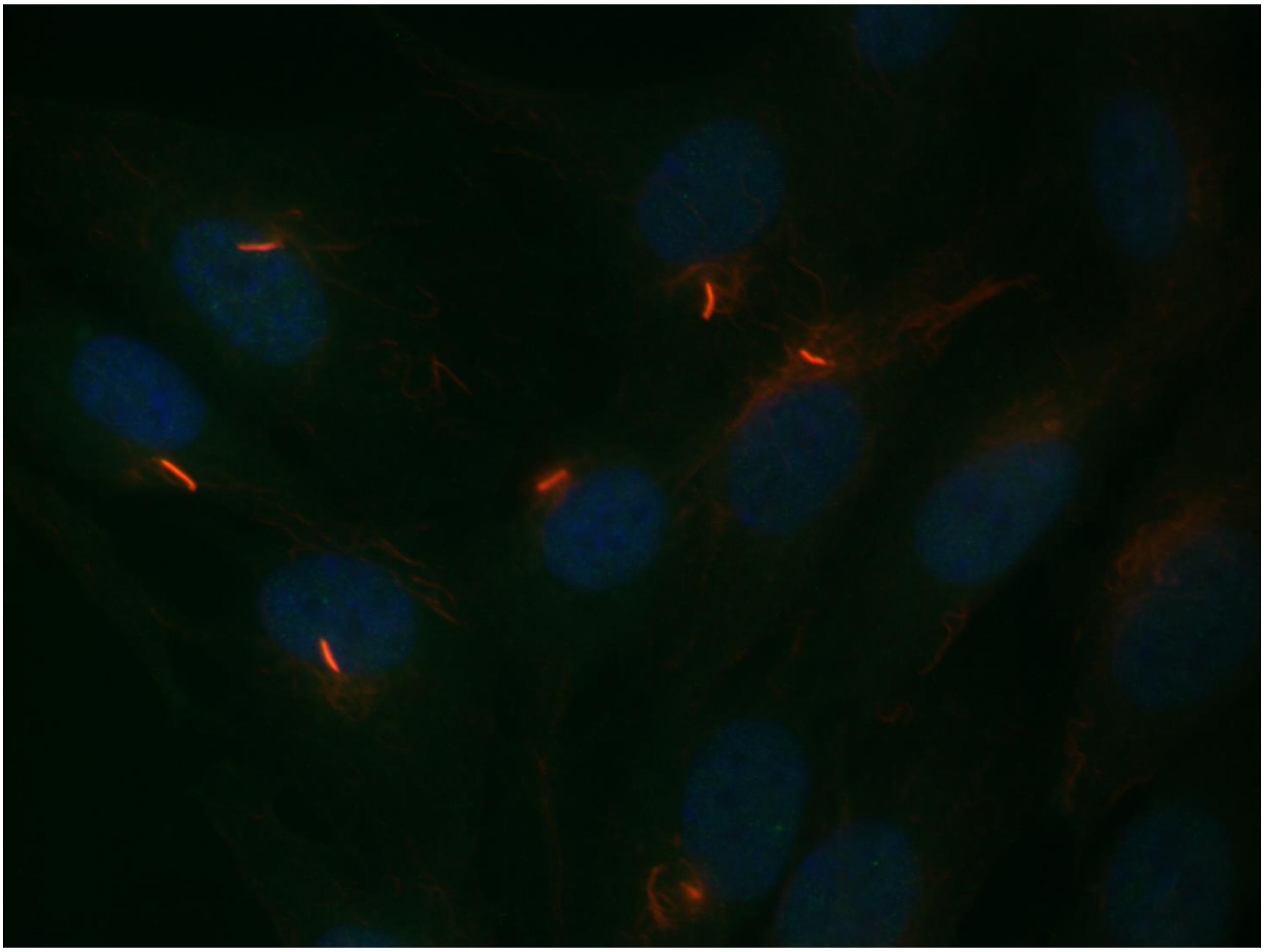

Ctrl + SAG 200 nM + tubacin

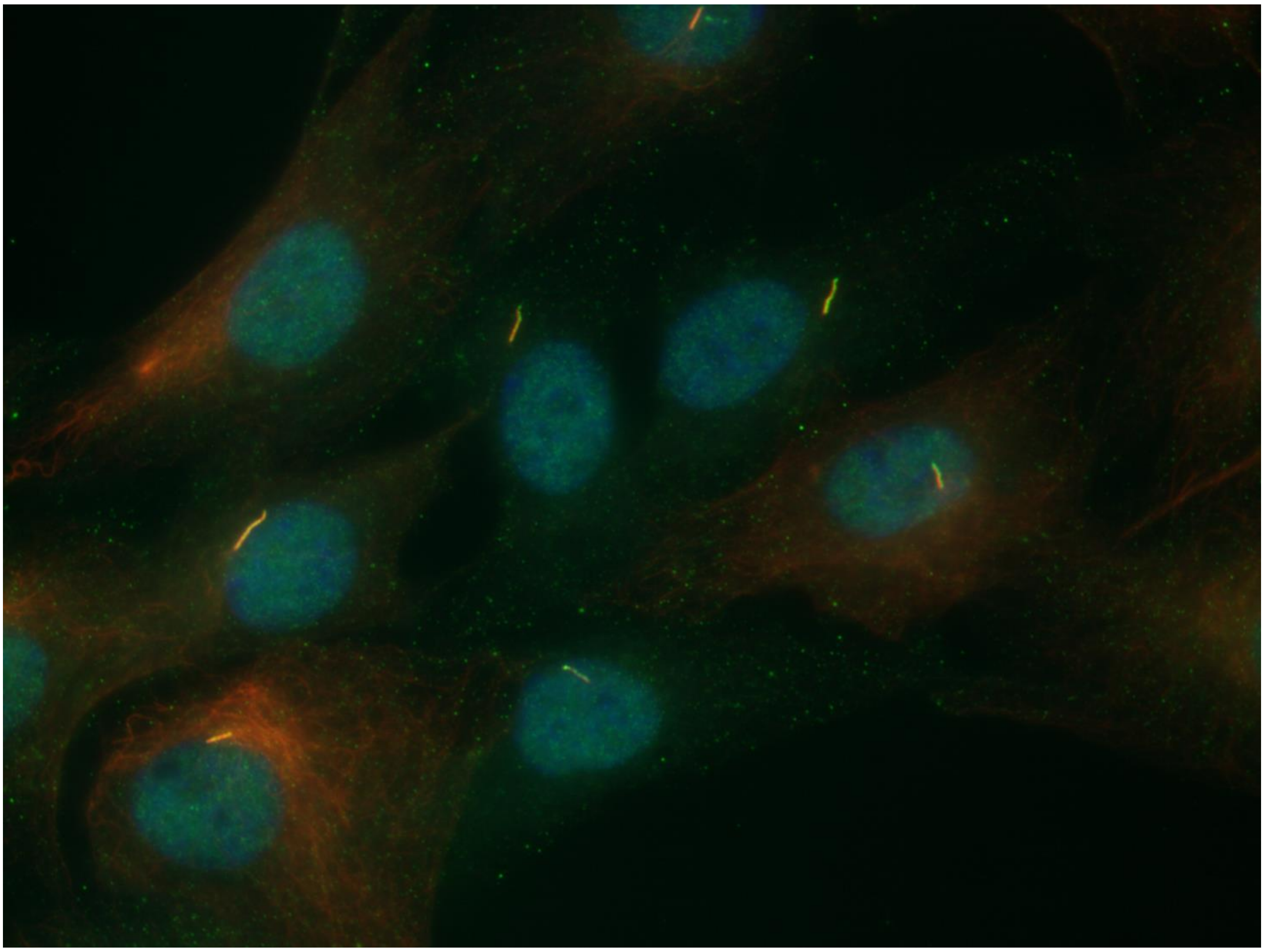

siMeCP2 not treated

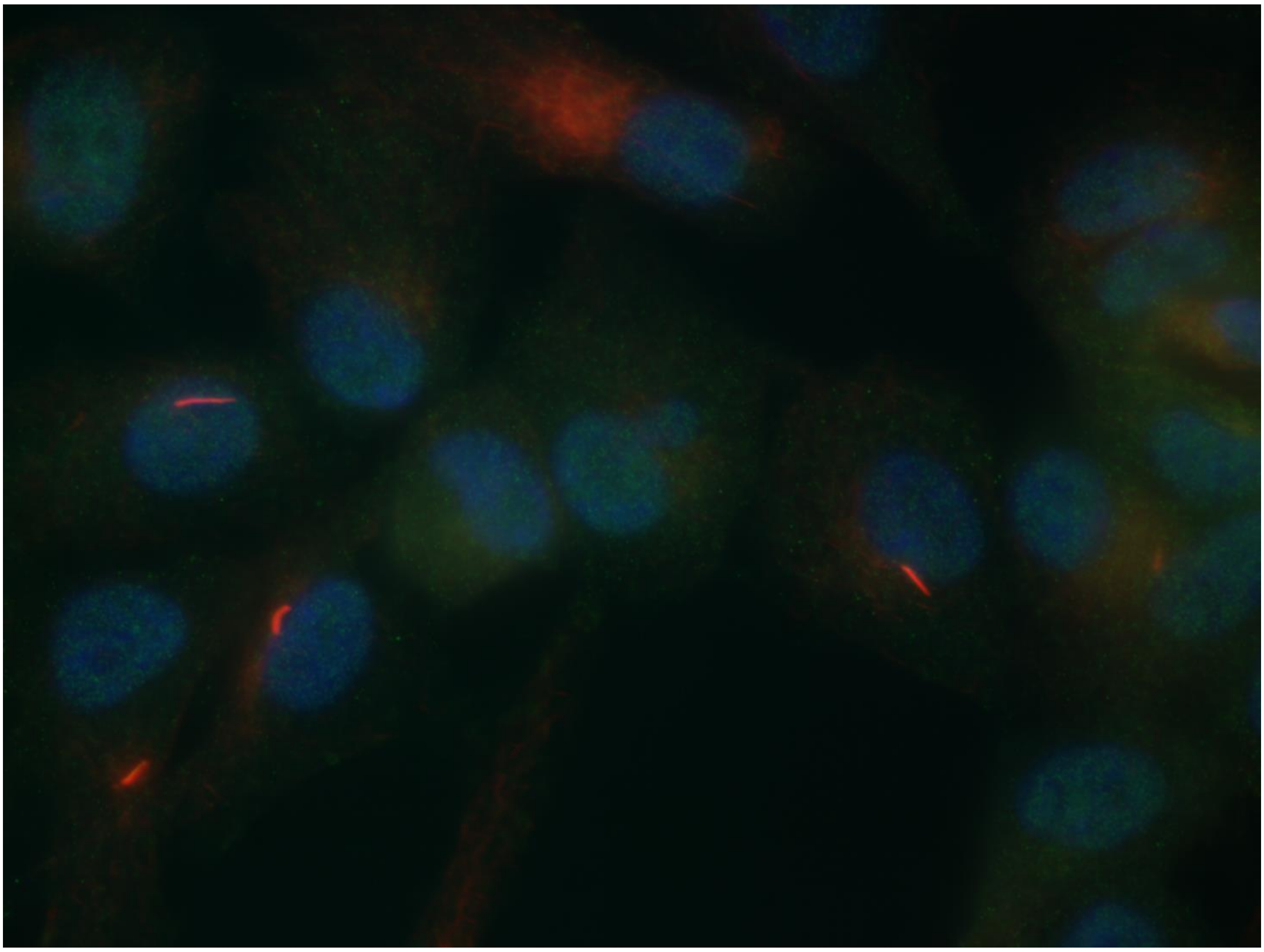

siMeCP2 + SAG 200nM

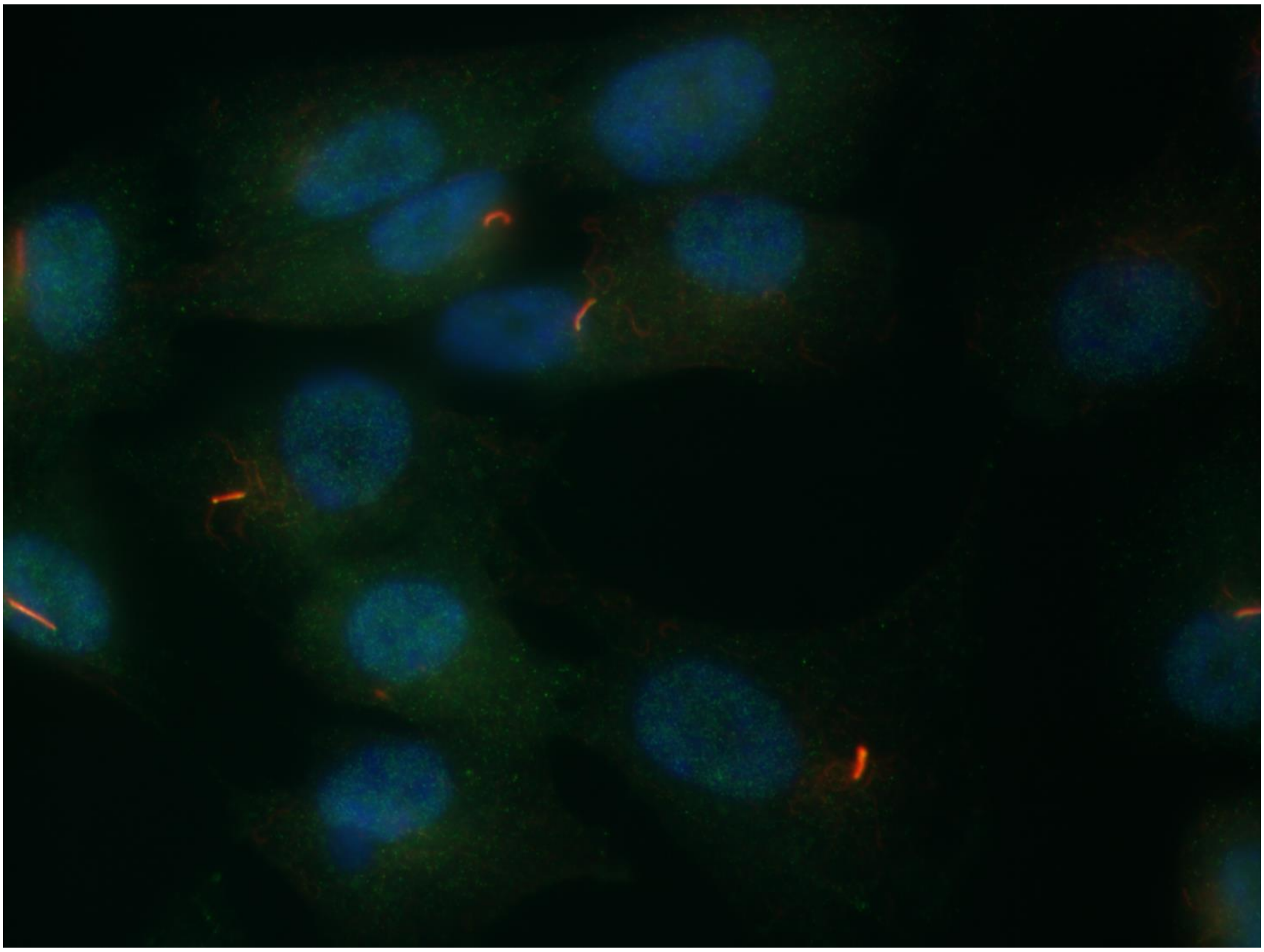

siMeCP2 + SAG + tubacin

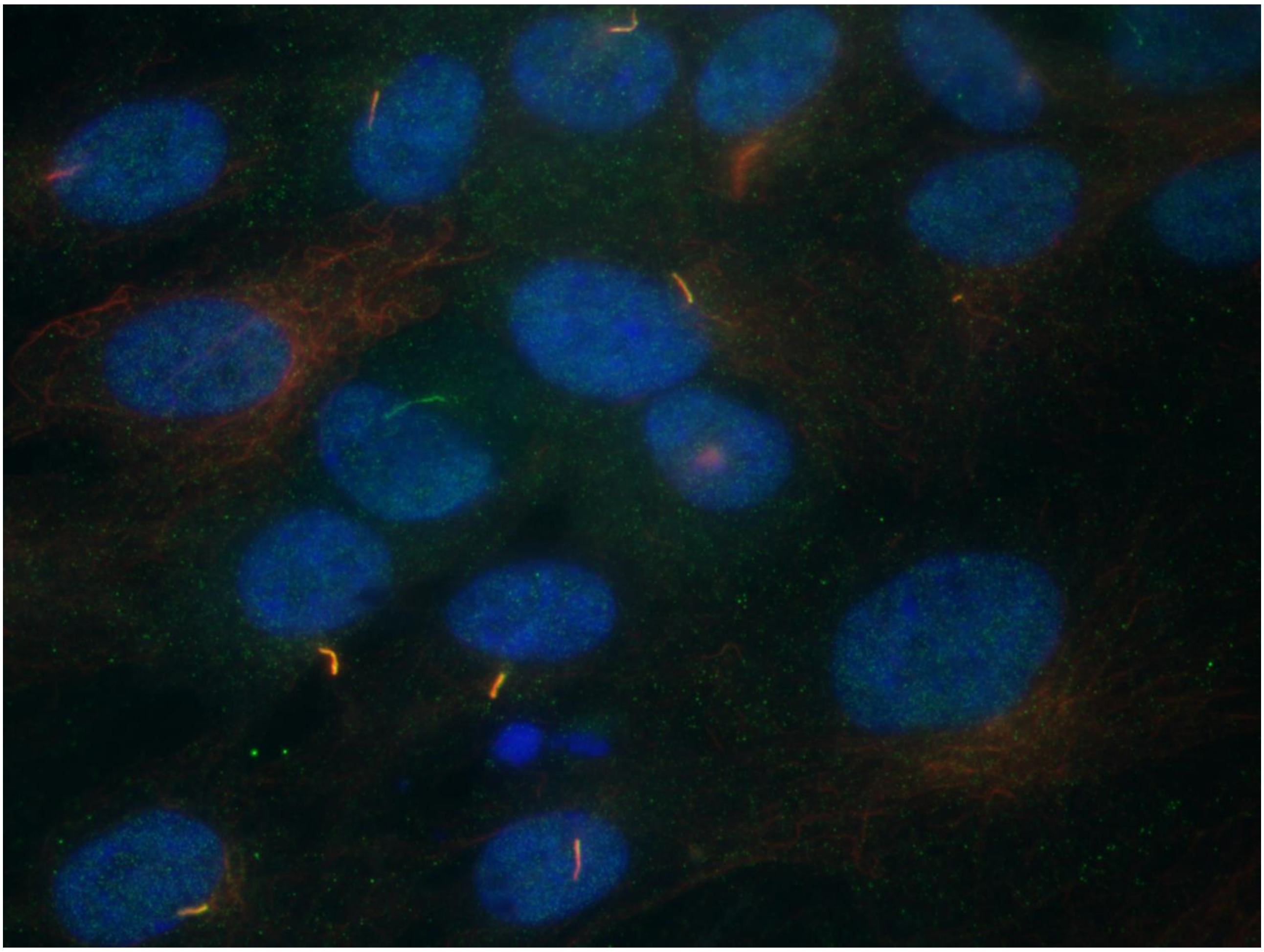

Supplement: Supplementary file 8 — Source Data for Figure 5 [file EMMM-12-e10270-s007.pdf]
