# Supplementary figures and images for: MECP2 mutations affect ciliogenesis: a novel perspective for Rett syndrome and related disorders
Source: EMBO Mol Med. 2020 May 8;12(6):e10270. doi: 10.15252/emmm.201910270 (PMC7278541; doi:10.15252/emmm.201910270)

Figure 6A

WT

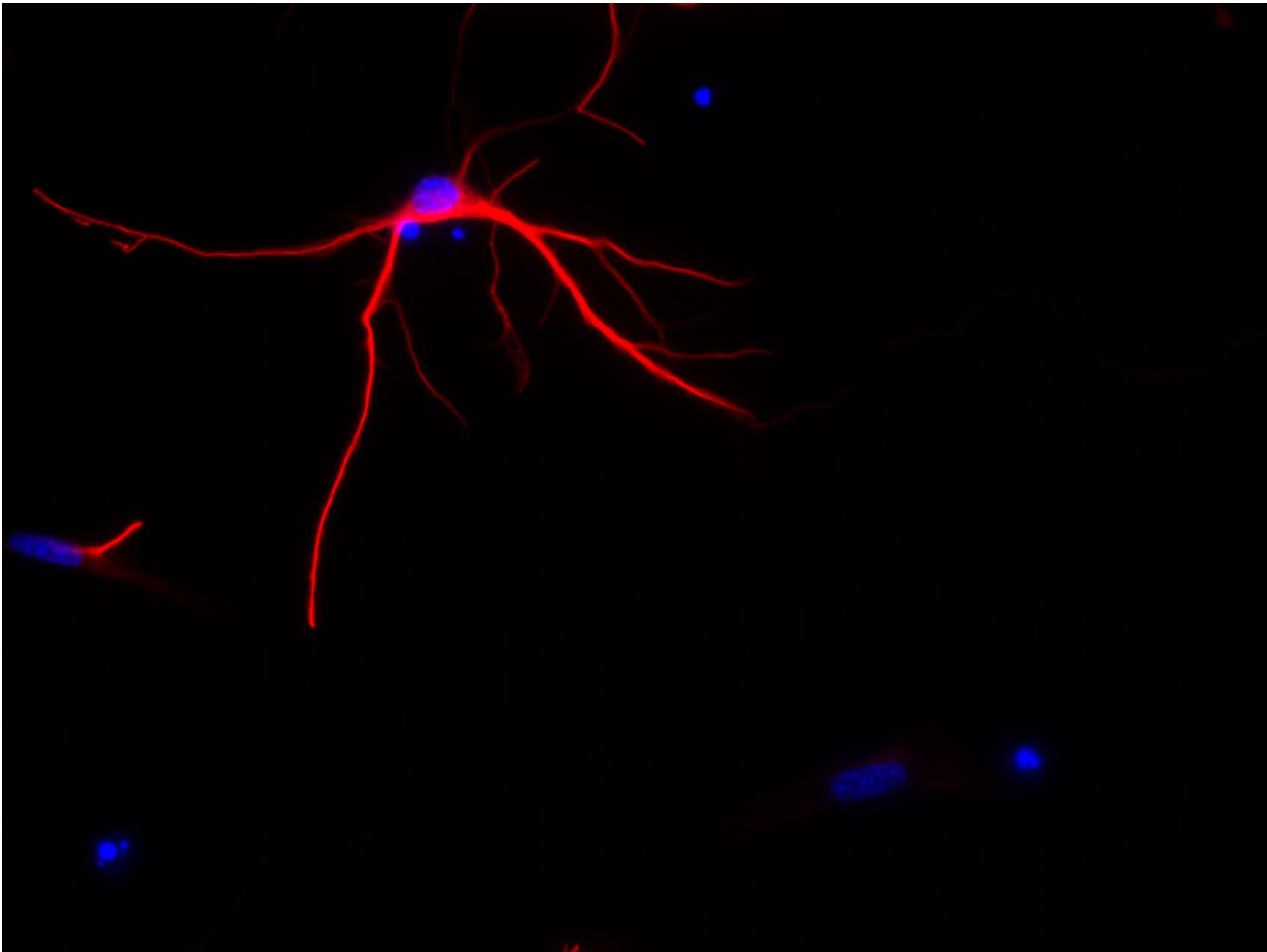

Mecp2 KO

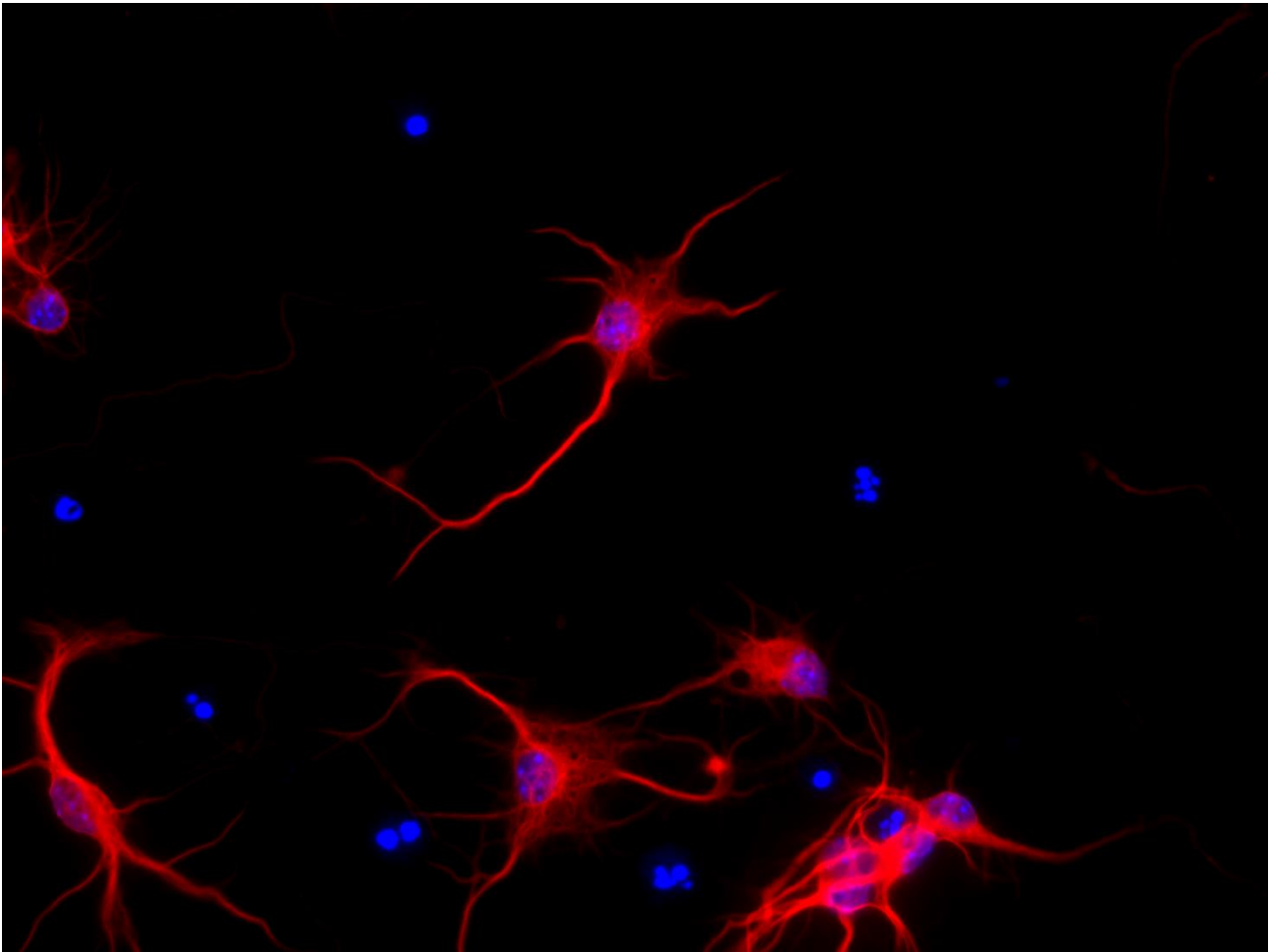

Mecp2 KO + tubacin

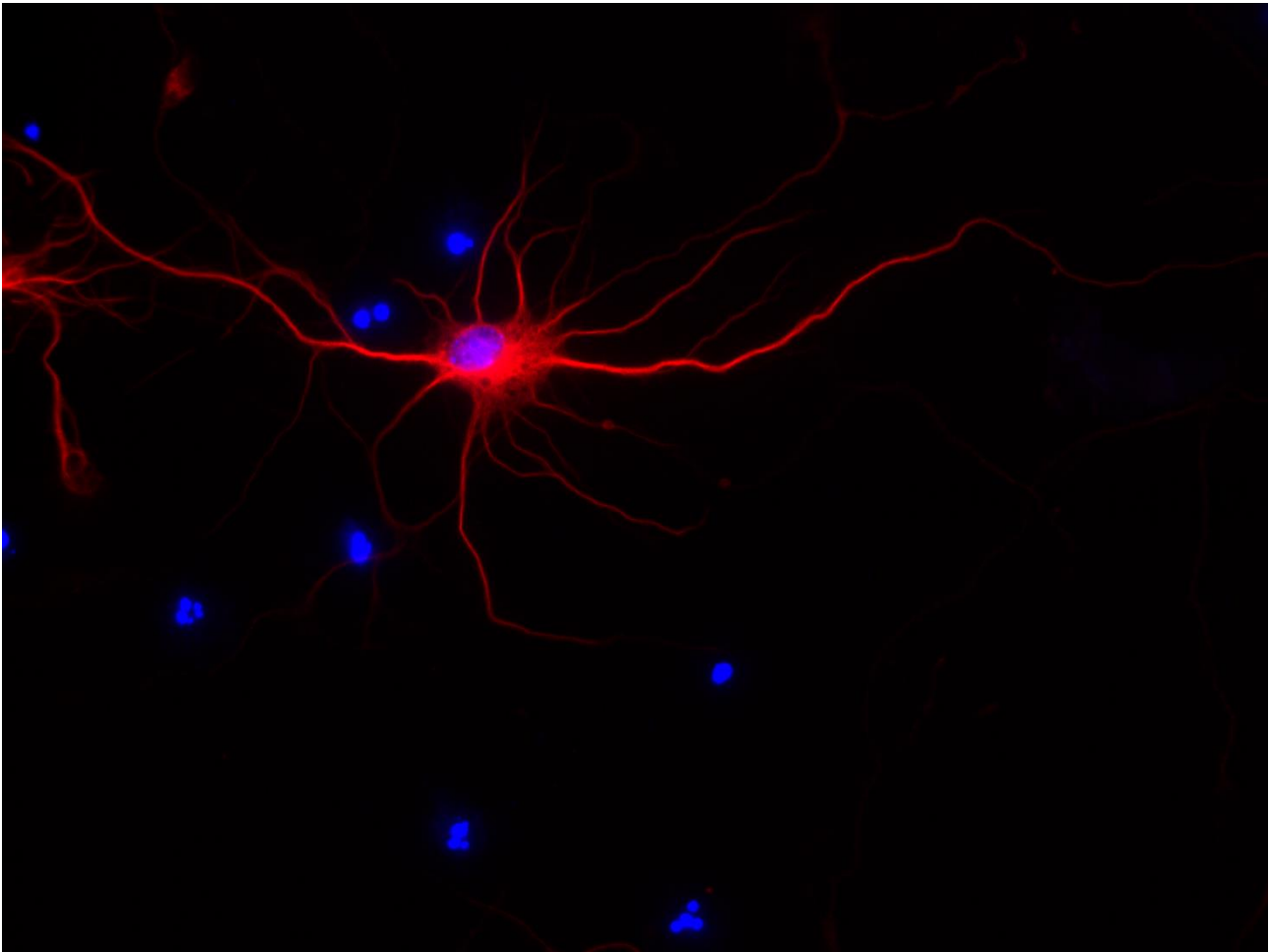

Mecp2 KO + TC-S7010

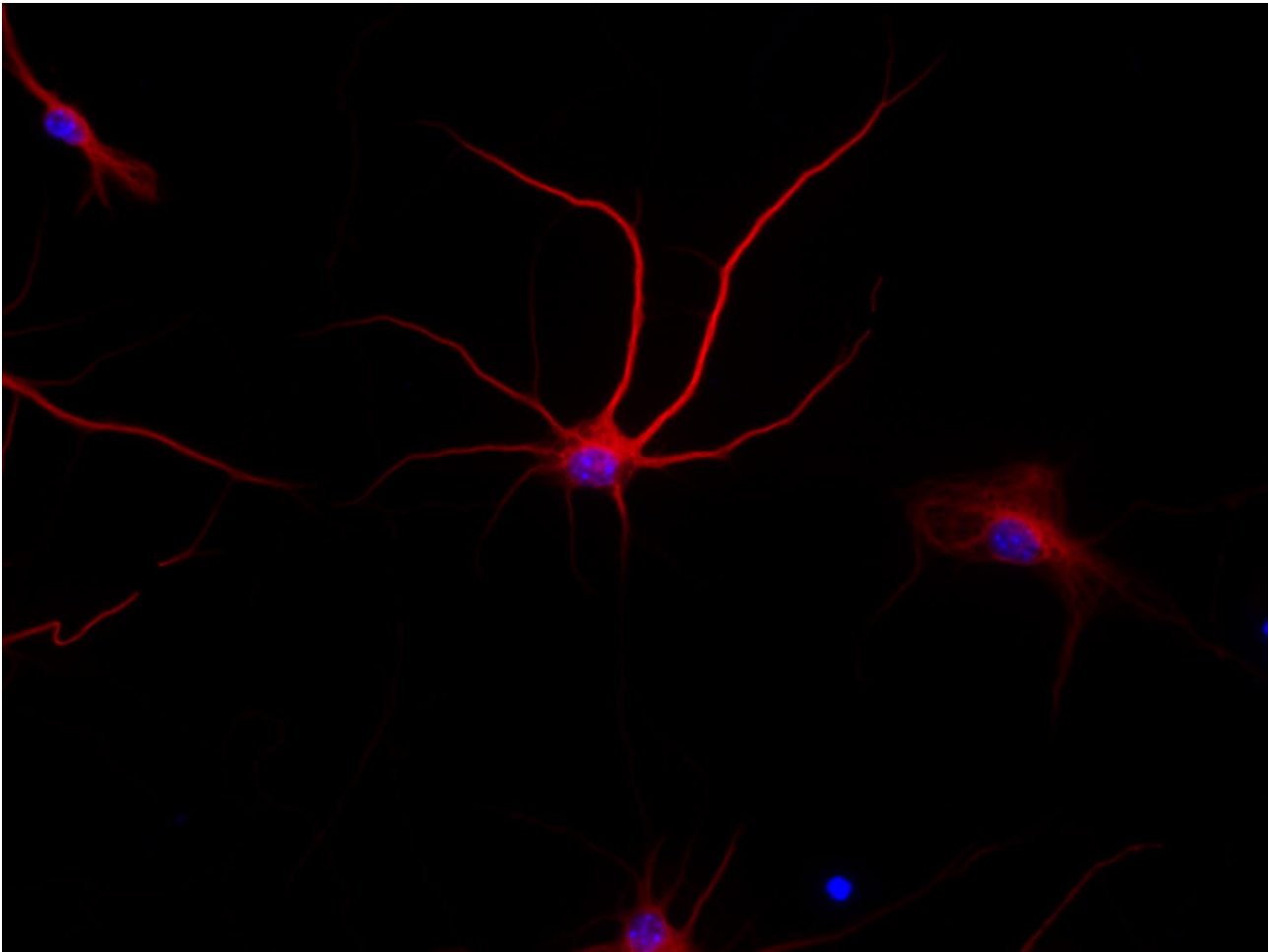

Figure 6C

WT

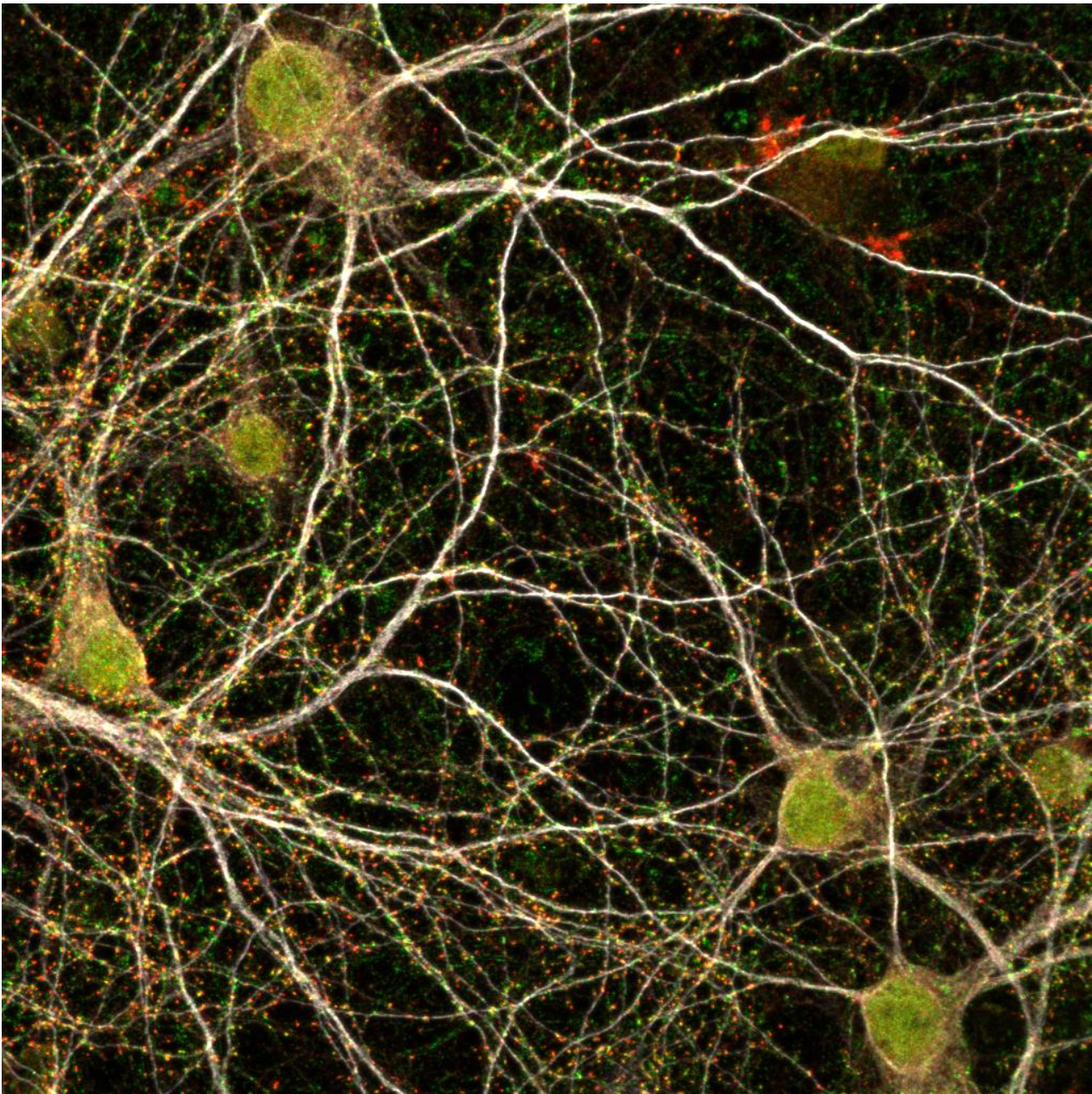

Mecp2 KO

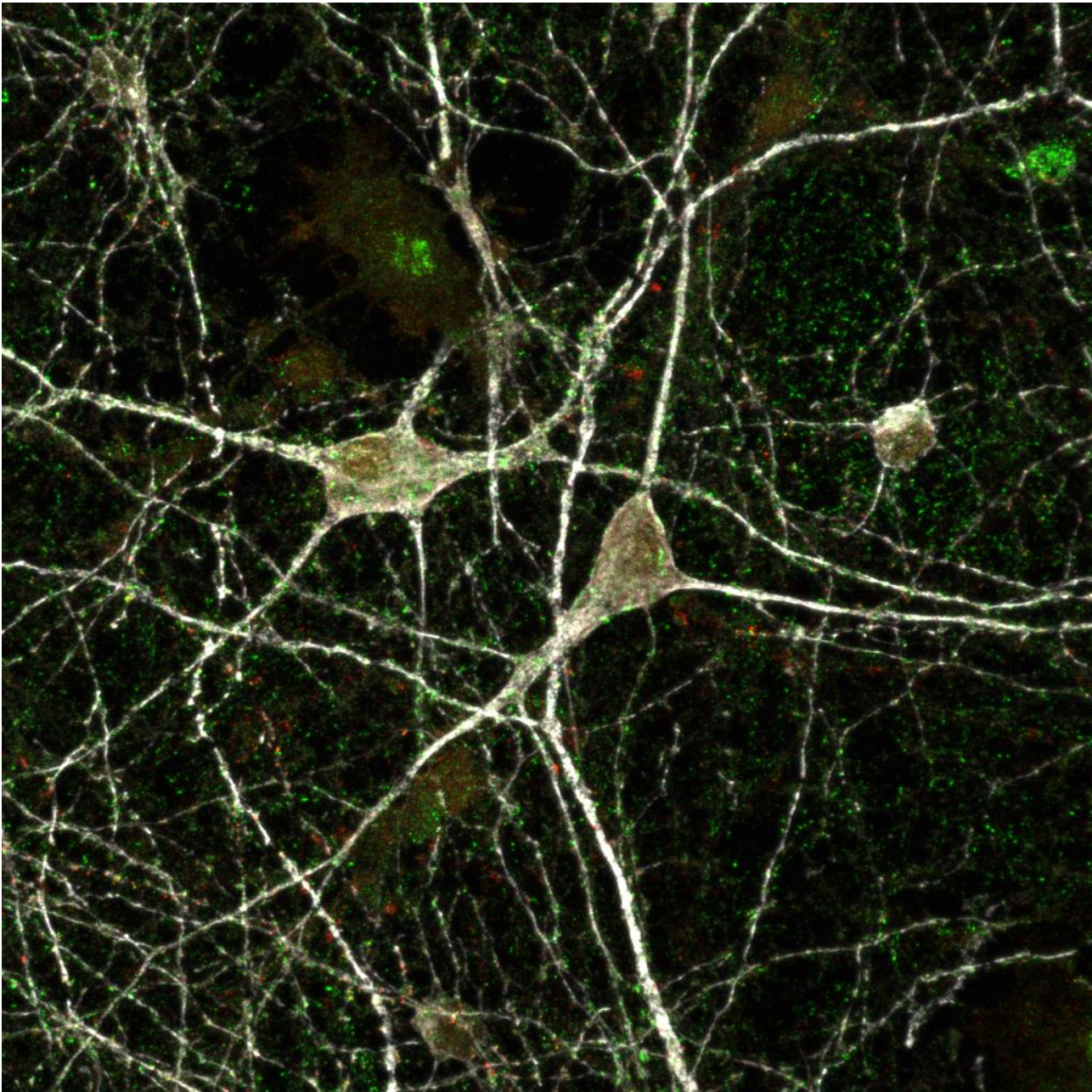

Mecp2 KO + tubacin

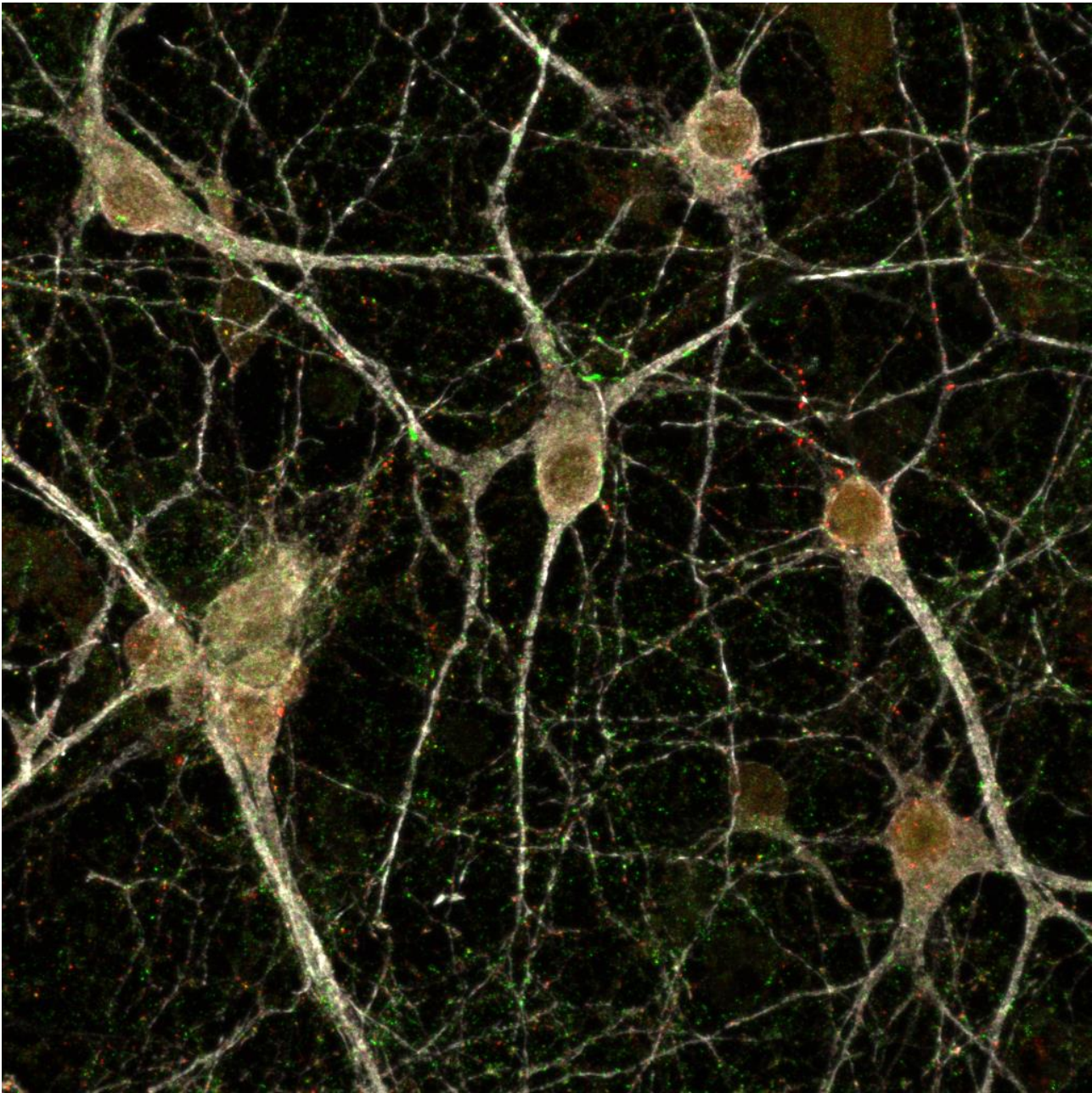

Mecp2 KO + TC-S7010

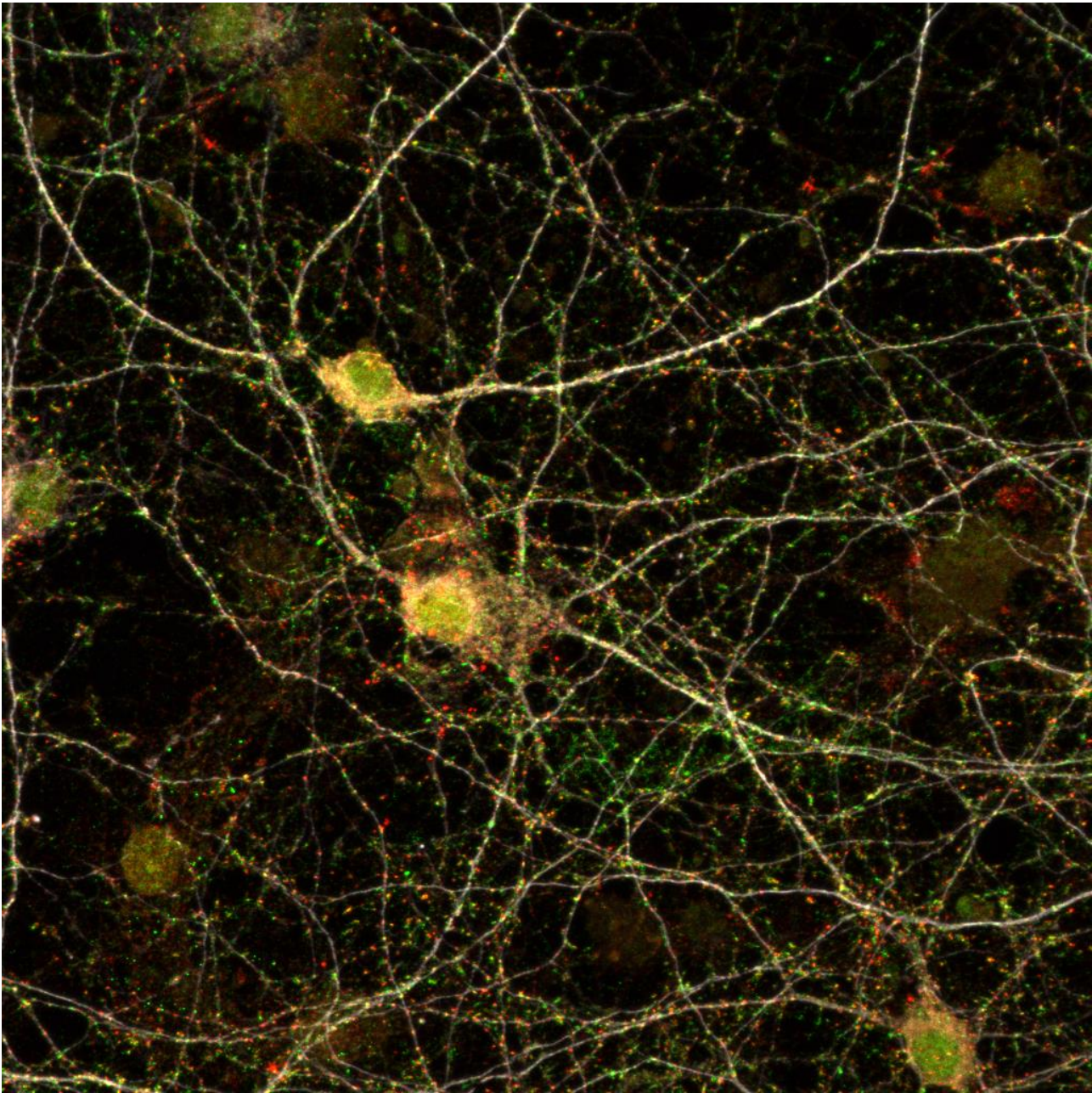

Supplement: Supplementary file 9 — Source Data for Figure 6 [file EMMM-12-e10270-s008.pdf]

**Figure 7A**

Ctrl fibroblasts

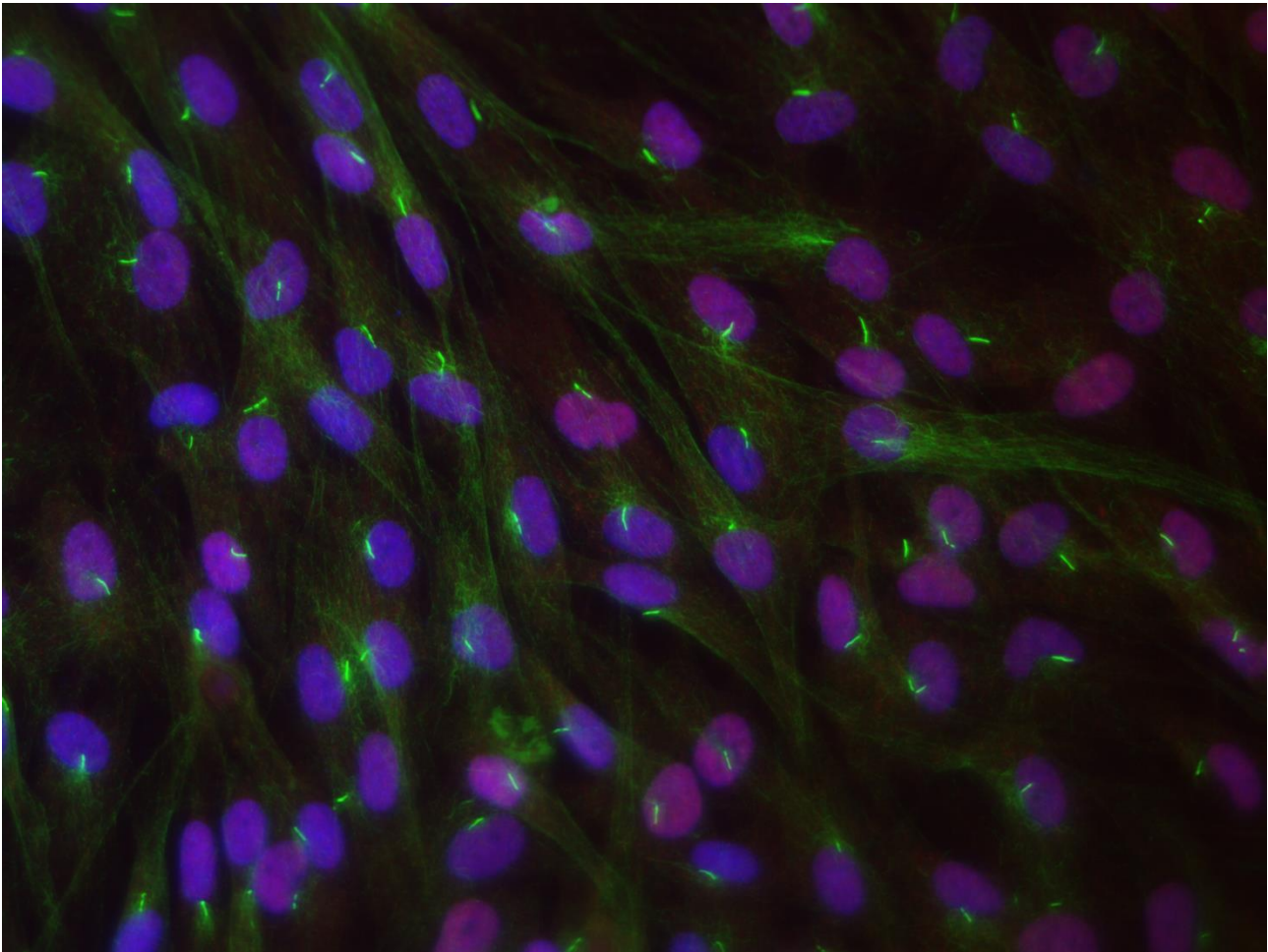

705delG

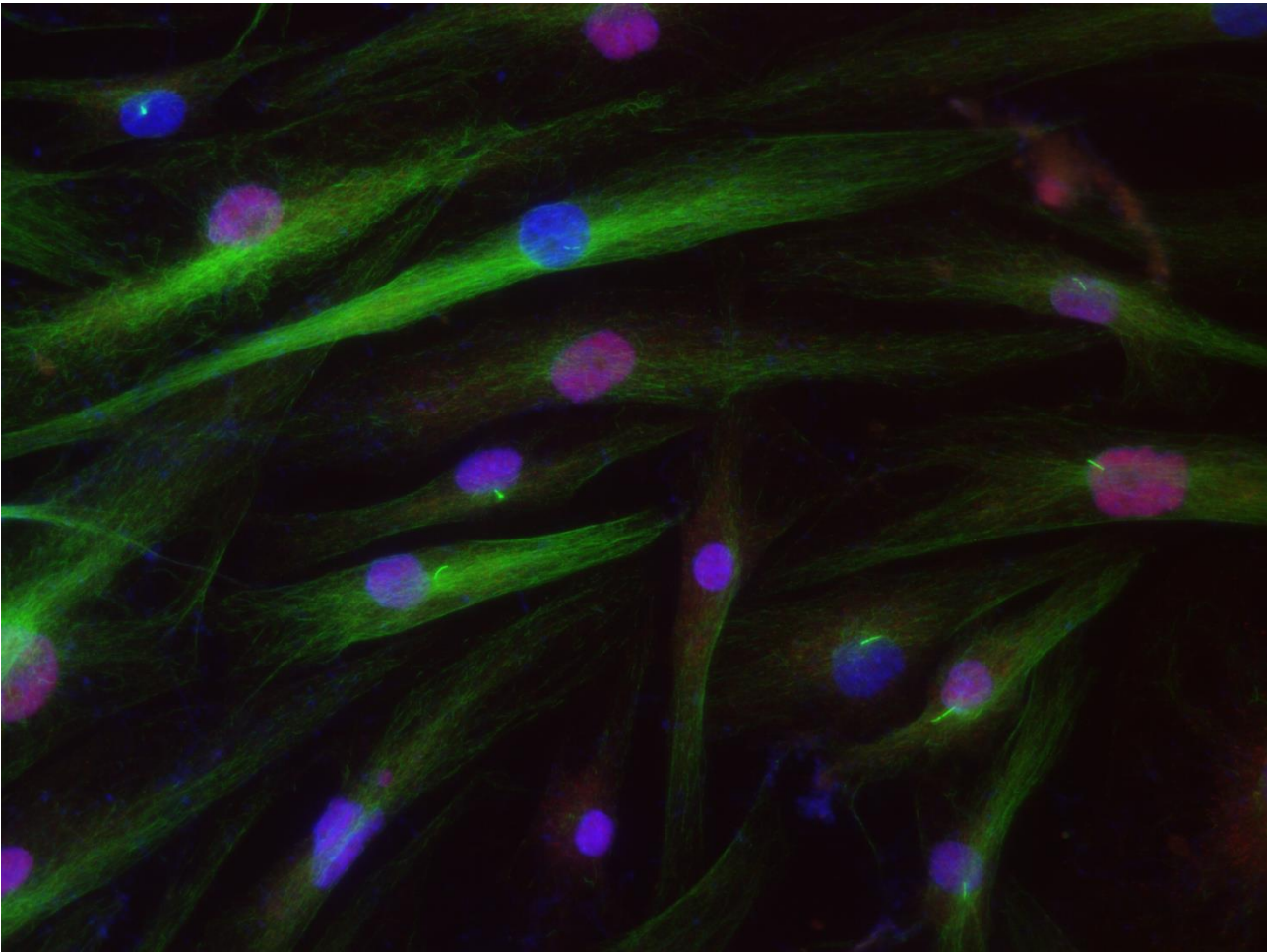

Q244X

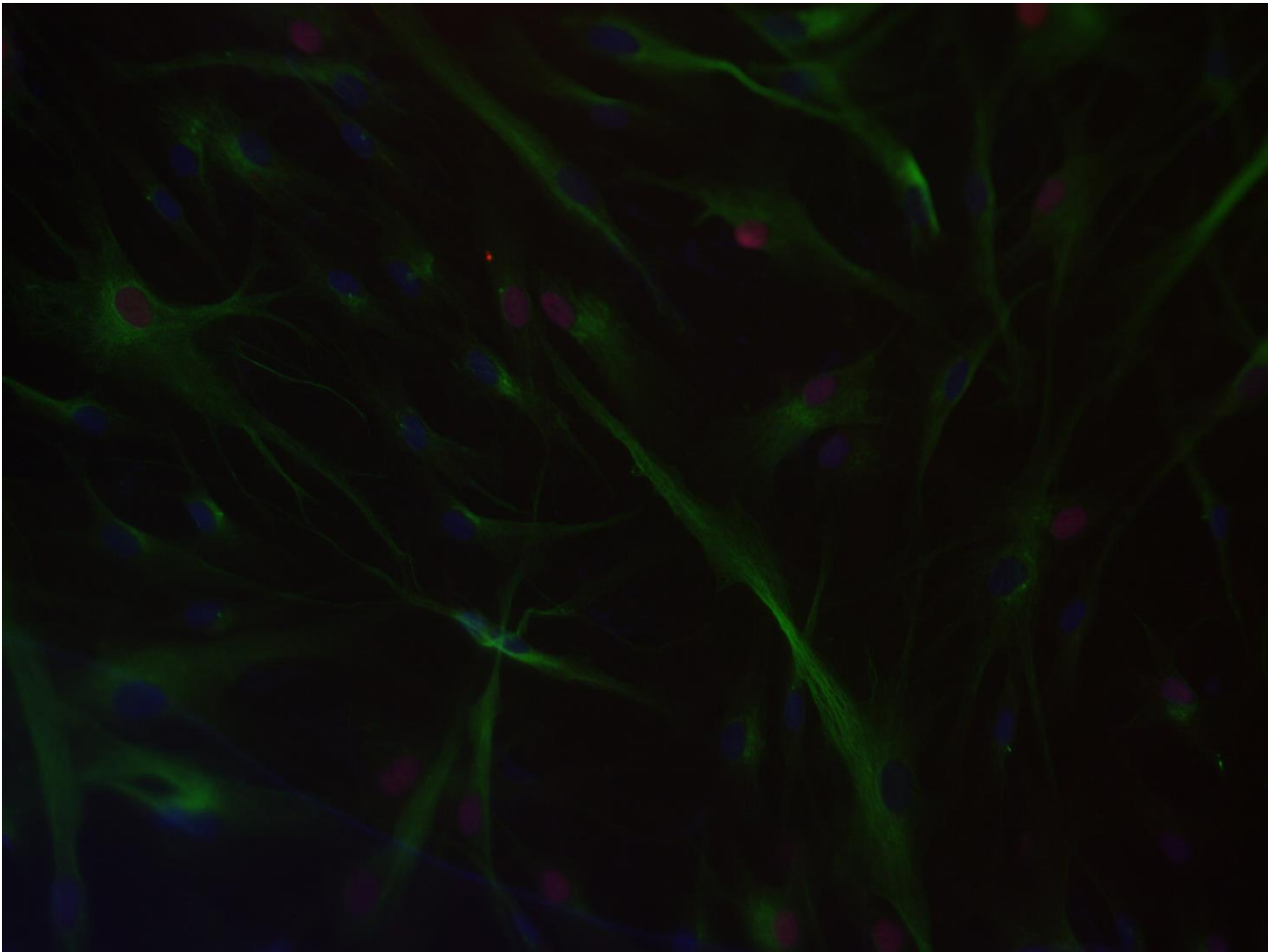

R255X

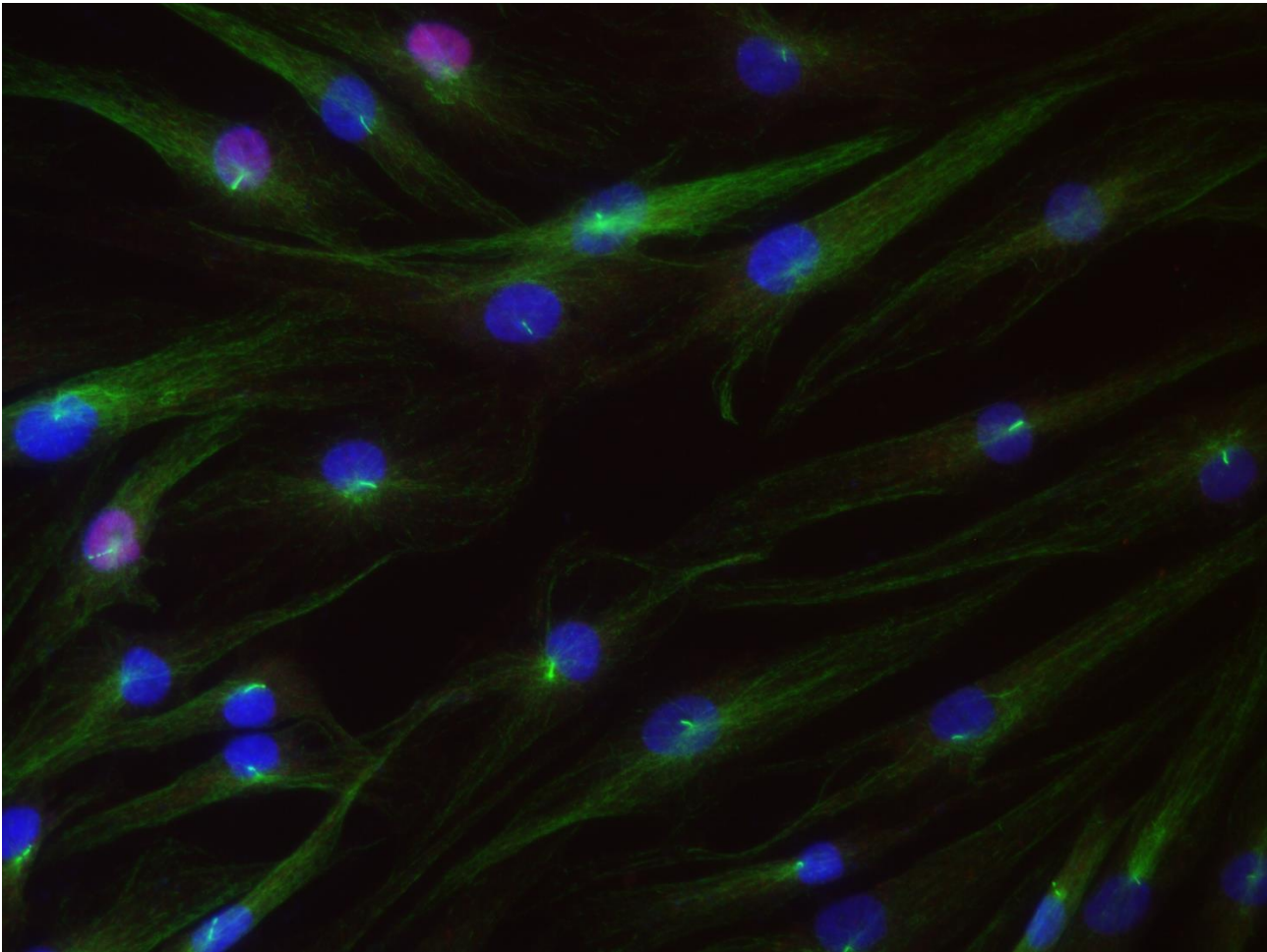

Supplement: Supplementary file 10 — Source Data for Figure 7 [file EMMM-12-e10270-s009.pdf]
